# Phosphorylation of KasB Regulates Virulence and Acid-Fastness in *Mycobacterium tuberculosis*

**DOI:** 10.1371/journal.ppat.1004115

**Published:** 2014-05-08

**Authors:** Catherine Vilchèze, Virginie Molle, Séverine Carrère-Kremer, Jade Leiba, Lionel Mourey, Shubhada Shenai, Grégory Baronian, Joann Tufariello, Travis Hartman, Romain Veyron-Churlet, Xavier Trivelli, Sangeeta Tiwari, Brian Weinrick, David Alland, Yann Guérardel, William R. Jacobs, Laurent Kremer

**Affiliations:** 1 Howard Hughes Medical Institute, Department of Microbiology and Immunology, Albert Einstein College of Medicine, Bronx, New York, New York, United States of America; 2 Laboratoire de Dynamique des Interactions Membranaires Normales et Pathologiques, Universités de Montpellier II et I, CNRS; UMR 5235, Montpellier, France; 3 Institut de Pharmacologie et de Biologie Structurale, CNRS, Toulouse, France; 4 The Université de Toulouse, Université Paul Sabatier, IPBS, Toulouse, France; 5 Division of Infectious Diseases, Department of Medicine, and the Ruy V. Lourenco Center for the Study of Emerging and Reemerging Pathogens, New Jersey Medical School, Rutgers Biomedical and Health Sciences, Newark, New Jersey, United States of America; 6 Université Lille 1, Unité de Glycobiologie Structurale et Fonctionnelle, UGSF, Villeneuve d'Ascq, France; 7 CNRS, UMR 8576, Villeneuve d'Ascq, France; 8 INSERM, DIMNP, Montpellier, France; University of Massachusetts, United States of America

## Abstract

*Mycobacterium tuberculosis* bacilli display two signature features: acid-fast staining and the capacity to induce long-term latent infections in humans. However, the mechanisms governing these two important processes remain largely unknown. Ser/Thr phosphorylation has recently emerged as an important regulatory mechanism allowing mycobacteria to adapt their cell wall structure/composition in response to their environment. Herein, we evaluated whether phosphorylation of KasB, a crucial mycolic acid biosynthetic enzyme, could modulate acid-fast staining and virulence. Tandem mass spectrometry and site-directed mutagenesis revealed that phosphorylation of KasB occurred at Thr334 and Thr336 both *in vitro* and in mycobacteria. Isogenic strains of *M. tuberculosis* with either a deletion of the *kasB* gene or a *kasB*_T334D/T336D allele, mimicking constitutive phosphorylation of KasB, were constructed by specialized linkage transduction. Biochemical and structural analyses comparing these mutants to the parental strain revealed that both mutant strains had mycolic acids that were shortened by 4–6 carbon atoms and lacked *trans*-cyclopropanation. Together, these results suggested that in *M. tuberculosis*, phosphorylation profoundly decreases the condensing activity of KasB. Structural/modeling analyses reveal that Thr334 and Thr336 are located in the vicinity of the catalytic triad, which indicates that phosphorylation of these amino acids would result in loss of enzyme activity. Importantly, the *kasB*_T334D/T336D phosphomimetic and deletion alleles, in contrast to the *kasB*_T334A/T336A phosphoablative allele, completely lost acid-fast staining. Moreover, assessing the virulence of these strains indicated that the KasB phosphomimetic mutant was attenuated in both immunodeficient and immunocompetent mice following aerosol infection. This attenuation was characterized by the absence of lung pathology. Overall, these results highlight for the first time the role of Ser/Thr kinase-dependent KasB phosphorylation in regulating the later stages of mycolic acid elongation, with important consequences in terms of acid-fast staining and pathogenicity.

## Author Summary

Acid-fast staining has been used since 1882 as the hallmark diagnostic test for detecting *Mycobacterium tuberculosis*, the causative agent of tuberculosis. It has been attributed to the presence of a waxy cell envelope, and primarily to its key components, mycolic acids. Here, we report a new mechanism of regulation in which phosphorylation of KasB, involved in the completion of full-length mycolic acids, leads to shortened mycolic acids and loss of acid-fast staining. Moreover, a *M. tuberculosis* mutant strain mimicking constitutive phosphorylation of KasB is severely attenuated for growth in both immunocompetent and immunosuppressed mice and fails to cause mortality and pathophysiological symptoms. These results emphasize the critical role of kinase-dependent phosphorylation in the pathogenesis of *M. tuberculosis* by controlling the mycolic acid chain length. Our study demonstrates the importance of a regulatory mechanism governing acid-fastness and virulence of *M. tuberculosis*.

## Introduction

*Mycobacterium tuberculosis* (*Mtb*) is an extraordinarily versatile pathogen that can exist in two distinct states in the host, leading to asymptomatic latent infection in which bacilli are present in a non-replicating dormant form, or to active tuberculosis (TB), characterized by actively replicating organisms. Establishment of these different (patho)physiological states requires mechanisms to sense a wide range of environmental signals and to coordinately regulate multiple metabolic and cellular processes. Many of the stimuli encountered by *Mtb* are transduced *via* transmembrane sensor kinases, allowing the pathogen to adapt to survive in hostile environments. In addition to the 12 classical two-component systems [1], *Mtb* contains 11 eukaryotic-like Ser/Thr protein kinases (STPK) [2], [3], suggesting that these two phospho-based signaling systems are of comparable importance in this microorganism. Knowledge of the substrates of each of the *Mtb* STPK is essential for understanding their function. Several kinase-substrates pairs have been identified and characterized during the last decade. In addition, a recent comprehensive understanding of *in vivo* phosphorylation event in *Mtb* was gained using a mass spectrometry-based approach to identify phosphorylation sites in *Mtb* proteins [4]. This provided insights into the range of functions regulated by Ser/Thr phosphorylation, underpinning the involvement of many STPK in regulating metabolic processes, transport of metabolites, cell division or virulence [5], [6], [7].

Recent studies focusing on mycolic acid biosynthesis regulation have shown that most essential enzymes forming the central core of type II fatty acid synthase (FASII) are phosphorylated by STPK [6] and that, at least *in vitro*, post-translational phosphorylation inhibits the activity of these enzymes. These include the β-ketoacyl ACP synthase KasA, the β-ketoacyl-ACP reductase MabA, the hydroxyacyl-ACP dehydratases HadAB and HadBC and the enoyl-ACP reductase InhA [8], [9], [10], [11], [12]. These studies culminated with the demonstration that InhA, also known as the primary target of the first-line anti-TB drug isoniazid (INH), is controlled *via* phosphorylation by STPK on Thr266 both *in vitro* and *in vivo* [10], [11]. The physiological relevance of Thr266 phosphorylation was demonstrated using *inhA* phosphoablative (T266A) or phosphomimetic (T266D/E) mutant strains. Not only was the enoylreductase activity severely impaired in the mimetic mutants *in vitro*, but introduction of *inhA*_T266D/E failed to complement an *inhA*-thermosensitive *M. smegmatis* strain, in agreement with mycolic acid inhibition, in a manner similar to that by isoniazid, and growth inhibition [10]. Altogether these results strongly suggest that *Mtb* may control in a very subtle manner its FASII system by regulating each step of the elongation cycle. Since phosphorylation of HadAB and HadBC enzymes was found to be increased during stationary growth phase, it was proposed that mycobacteria shut down meromycolic acid chain production under non-replicating conditions, a view which is supported by the fact that the mycolic acid biosynthesis is growth phase-dependent and is not functional during the stationary phase [13]. However, whether phosphorylation of FASII components may also directly participate in *Mtb* virulence, through the control of the meromycolic acid chain length has not been reported yet.

It was previously demonstrated that targeted deletion of *kasB*, one of two *Mtb* genes encoding distinct β-ketoacyl-ACP synthases, results in loss of acid-fast staining and synthesis of shorter mycolates [14]. Perhaps the most striking effect of *kasB* deletion was the ability of the mutant strain to persist in infected immunocompetent mice without causing disease or mortality. This indicated that KasB participates in the latest elongation steps by adding the last few carbon atoms to the growing acyl-ACP chains and plays a critical role in controlling *Mtb* physiopathology. From these data, it could be inferred that KasB activity may be tightly regulated in order to control the mycolic acid chain length during the infection process. We have previously reported that, like KasA, KasB was a substrate of STPK [8], suggesting that phosphorylation may represent a mechanism governing KasB activity and, as a consequence, mycolic acid chain length and *Mtb* virulence.

This prompted us to decipher an original mechanism linking post-translational phosphorylation of KasB with *Mtb* virulence in a mouse infection model. Herein, we demonstrate that phosphorylation of KasB on Thr334 and Thr336 dramatically alters the mycolic acid chain length and acid-fast staining. Importantly, a KasB phosphomimetic mutant of *Mtb* was found to be extremely attenuated in mice infection models. These results provide, for the first time, insights into the contribution and importance of FASII phosphorylation *in vivo* in the control of i) the clinically important feature of acid-fast staining in *Mtb* and ii) the physiopathology of TB.

## Results

### KasB is phosphorylated *in vivo* and *in vitro* on Thr334 and Thr336

**Figure 1:**
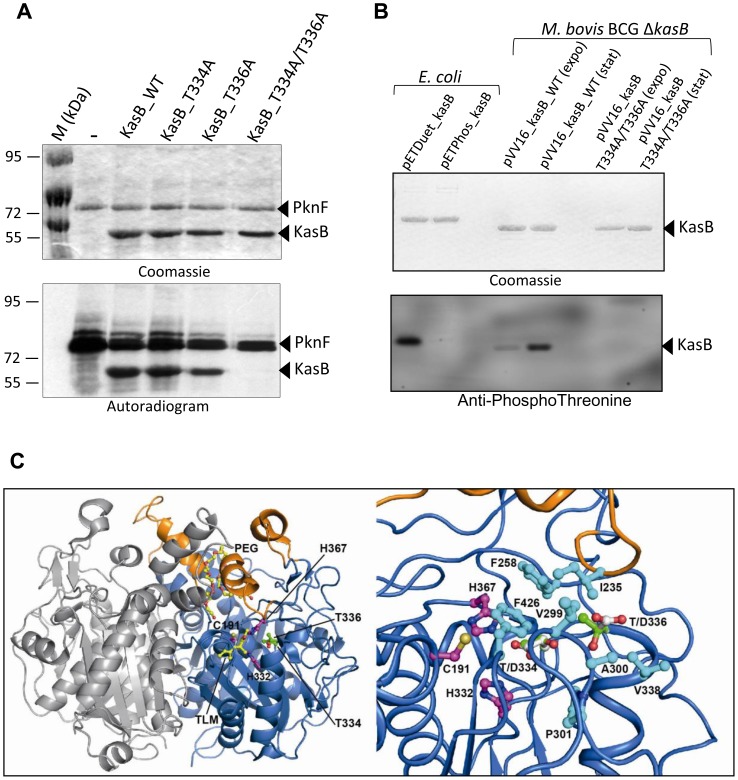
*M. tuberculosis* KasB is phosphorylated on Thr334 and Thr336. (A) *In vitro* phosphorylation of KasB with PknF. The PknF kinase encoded by the *Mtb* genome was expressed and purified as a GST fusion and incubated with purified His-tagged KasB_WT, KasB_T334A, KasB_T336A and KasB_T334A/T336A in the presence of radiolabeled [γ-^33^]ATP. Samples were separated by SDS-PAGE, stained with Coomassie Blue and visualized by autoradiography after overnight exposure to a film as indicated. Upper bands reflect the autophosphorylation activity of PknF whereas the lower bands correspond to the phosphorylation signal of KasB. **(B)** ***In vivo*** **phosphorylation of KasB.** A *kasB* deletion mutant of *M. bovis* BCG was transformed with either pVV16_kasB_WT or pVV16_kasB_T334A/T336A and grown in Sauton medium. Exponential (expo) or stationary (stat) phase cells were harvested, lyzed and processed for KasB purification by affinity chromatography on Ni^2+^-containing beads. The BCG-derived KasB_WT and KasB_T334A/T336A proteins were separated by SDS-PAGE, either stained with Coomassie Blue (upper panel) or subjected to Western blot analysis after probing the membrane with anti-phosphoThreonine antibodies (lower panel). Specificity of phosphothreonine recognition was checked by probing the antibodies against recombinant KasB produced in *E. coli* strains carrying either pETPhos_kasB (non phosphorylated KasB) or pETDuet_kasB that co-expresses KasB with PknF (phosphorylated KasB). **(C) Localization of Thr334 and Thr336 phospho-sites in the three-dimensional structure of KasB.** Overall view **(left panel)** showing the KasB dimeric structure ([19]; PDB entry 2GP6) in ribbon representation with the core domain in *marine* and the cap domain in *orange*. The second chain of the dimer is in *light gray*. The Cys-His-His catalytic triad and the two phospho-sites are displayed as ball-and-stick with carbon atoms in *magenta* and *green*, respectively. Also shown with carbon atoms in yellow are the TLM inhibitor and the PEG molecule as observed in the structure of the KasA C171Q acyl enzyme mimic ([20]; PDB entry 2WGG). The PEG molecule is thought to delineate the acyl-binding channel [20]. Nitrogens are in *blue*, oxygens in *red*, and sulfur in *gold*. Close-up view **(right panel)** after a 45° rotation of the left panel along a vertical axis. Side-chains of residues delineating the active site hydrophobic tunnel and of aspartic residues at positions 334 and 336 were also represented (carbons in *cyan* and *white*, respectively).

Previous work demonstrated that KasB is a substrate for several Ser/Thr protein kinases with PknF being one of the most efficient kinase [8]. However, the role and contribution of KasB phosphorylation with respect to the *Mtb* physiology and pathogenicity remains unknown, mainly because of the lack of information regarding the identity of the phosphoacceptors. Therefore, recombinant wild-type KasB (unphosphorylated form) was expressed and purified from *E. coli* harboring pETPhos_kasB and used in an *in vitro* kinase assay in the presence of PknF and [γ-^33^P]ATP. The reaction mixture was then separated by SDS-PAGE and analyzed by autoradiography, revealing a specific band corresponding to the phosphorylated form of KasB (Fig. 1A), as reported previously [8]. To identify the number and nature of the phosphosites, the protein was phosphorylated *in vitro* with PknF and cold ATP and subjected to mass spectrometry analysis after tryptic and chymotryptic digestions, a method successfully used to elucidate the phosphoacceptors in a sequence-specific fashion for several other *Mtb* STPK substrates [7], [9], [10], [15], [16], [17]. Spectral identification and phosphorylation determination were achieved with the paragon algorithm from the 2.0 database-searching software (Applied Biosystems) using the phosphorylation emphasis criterion against a homemade database that included the sequences of KasB and derivatives. The sequence coverage was 92% with the non-covered sequence free of serine or threonine residues. Phosphorylation was detected only on a single peptide _315_ AIQLAGLAPGDIDHVNAHATGTQVGDLAEGR_345_. The MS/MS spectra unambiguously confirmed the presence of two phosphate groups on this peptide (data not shown). The 315–345 peptide possesses only two Thr residues representing the potential phosphoacceptors, Thr334 and Thr336. Definitive identification of the phosphosites was achieved by site-directed mutagenesis, by replacing Thr with Ala, preventing subsequent phosphorylation. The single (T334A, T336A) and double (T334A/T336A) mutants were expressed *via* the pETPhos, purified as His-tagged proteins and individually subjected to the kinase assay. As shown in Fig. 1A, decrease of the phosphorylation signal was only partially limited in the single mutants with respect to the wild-type protein. However, phosphorylation was abrogated in the double mutant as evidenced by the absence of a specific radioactive band, confirming that, *in vitro*, phosphorylation of KasB occurs at Thr334 and Thr336. Similar results were obtained when KasB_T334A/T336A was incubated in the presence of either PknA, PknB, PknD, PknH or PknL, indicating that these two residues are the phosphoacceptor for all six kinases (Figure S1 in Text S1).

*In vivo* phosphorylation of KasB was next investigated in recombinant *M. bovis* BCG by Western blotting using anti-phosphothreonine antibodies. Specificity of the antibodies was first assessed against KasB purified from either *E. coli* (pETPhos_*kasB*) or *E. coli* co-expressing PknF (pETDuet_*kasB*), thanks to a recently developed duet strategy [18]. Phosphorylated KasB derived from pETDuet_*kasB* was specifically revealed with anti-phosphothreonine antibodies, while the unphosphorylated isoform from pETPhos_*kasB* failed to react (Fig. 1B), confirming the specificity of Thr phosphorylation. To confirm the phosphorylation status of KasB in mycobacteria, and in order to exclude eventual interference/association between the endogenous and the recombinant His-tagged KasB monomers (KasB being a dimer, see below), a Δ*kasB* BCG mutant was transformed with pVV1::*kasB*_WT or pVV16::*kasB*_T334A/T336A in which the wild-type or phosphoablative *kasB* genes were placed under the control of the *hsp60* promoter. BCG carrying either pVV16::*kasB*_WT or pVV16::*kasB*_T334A/T336A was harvested from exponential or stationary cultures prior to protein purification by affinity chromatography on Ni^2+^-containing agarose beads and Western blotting using phosphothreonine antibodies. Specific phosphorylation was only detected for the wild-type but not the T334A/T336A protein (Fig. 1B). The lack of reactivity with the double Ala mutant excludes the possibility of additional phosphorylation sites. Interestingly, phosphorylation of KasB was more pronounced during stationary phase than exponentially-growing bacteria, indicating that KasB phosphorylation is growth phase-dependent. Taken collectively, these data suggest that phosphorylation occurs at Thr334 and Thr336, *in vitro* and *in vivo*.

### Thr334 and Thr336 are in the vicinity of the catalytic triad

The crystal structure of KasB (438 residues, MW 46.4 kDa) in its apo-form has been determined to 2.4 Å resolution [19]. It consists of a dimer with each protomer adopting the typical thiolase fold decorated with specific structural features in the form of a cap (Fig. 1C). The structures of wild-type KasA (416 residues, MW 43.3 kDa), the other fatty acyl elongation β-ketoacyl synthase, and of the acyl enzyme mimic C171Q, both unliganded and with bound thiolactomycin (TLM), were also resolved to high resolution [20]. In line with their high sequence homology, KasA and KasB are structurally similar and superposition of the wild-type apo-dimers (PDB codes 2WGD and 2GP6, respectively) led to a root mean square deviation value of 1.1 Å for 814 aligned Cα atoms sharing 66% sequence identity. The active site, containing the Cys-His-His catalytic triad, is located in the core domain. As shown for KasA [20], TLM binds close to the active site in the malonyl-binding pocket and the hydrophobic acyl-binding channel of the substrate is connected to the malonyl-binding pocket and also directly accessible from the surface of the protein (Fig. 1C, left panel). Thr334 and Thr336 together with Ile235, Phe258, Val299, Ala300, Val338, Pro301, and Phe426 (most of these residues being strictly conserved in KasA) line a molecular tunnel that leads to the catalytic cysteine [19] (Fig. 1C, right panel). Thr334 is located close to the tunnel aperture whereas Thr336 directly faces the catalytic triad. Their side chains are aligned when looking from outside the tunnel with their OG atoms at a distance of 3.6 Å. Replacement of Thr334 and Thr336 by alanine would result in a slight broadening of one side of the tunnel. In contrast, Thr→Asp replacements that mimic constitutive phosphorylation [9], [10], [17], [21] are very likely to induce a profound perturbation in terms of steric hindrance and electrostatic potential. In addition, the carboxyl group of the Asp at position 334 could establish hydrogen bonds with the NE2 atoms of the two catalytic histidines. Thus, the perturbation brought by the Thr→Asp substitution might lead to severe impairment of the enzyme activity but not of the three-dimensional structure. Moreover, this was confirmed by analysis of the trypsinolysis kinetics of wild-type and mutated KasB proteins (Figure S2 in Text S1), and was consistent with the structural analysis indicating that introduction of Asp or Ala at position 334 and 336 does not seem to modify the folding of the protein as the proteolysis profiles of the different KasB derivatives were identical to the wild-type protein.

### Loss of acid-fast staining in a *M. tuberculosis* phosphomimetic mutant

**Figure 2:**
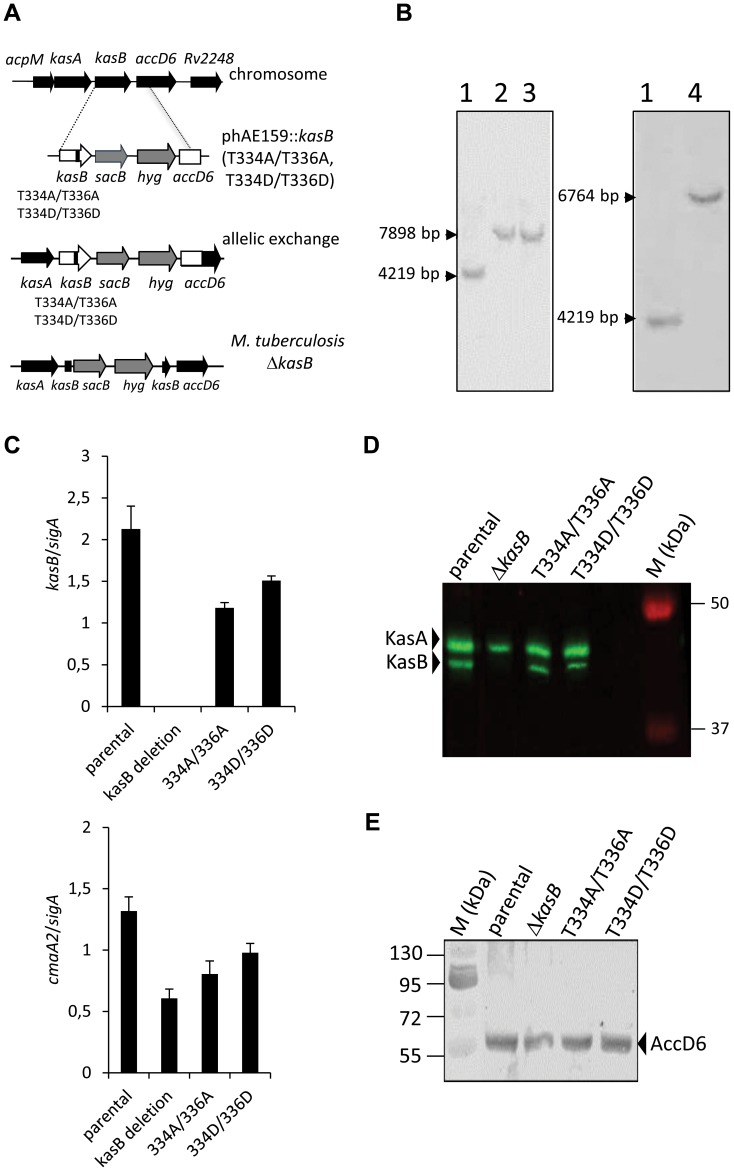
Construction of isogenic *M. tuberculosis* CDC1551 strains bearing the phosphoablative or phosphomimetic *kasB* alleles. (A) Schematic representation of the specialized transduction phage. A replicating shuttle phasmid derivative of phAE159 containing *kasB* carrying the mutations T336A/T336A or T334D/T336D, *sacB*, a *hyg* resistance cassette, and the first 959 bp of *accD6* was used to transduce *Mtb* CDC1551. If recombination occurs before the point mutation in *kasB*, this results in recombinant strains carrying the T334A/T336A or T334D/T336D mutations. The transductants were selected on hygromycin and screened by PCR amplification of *kasB* and presence of the desired mutations was confirmed by sequencing. **(B) Southern blot analysis of** ***M. tuberculosis kasB*** **mutant strains.** Genomic DNA from each strain was extracted, digested with *Bgl*II and analyzed: left panel, Southern for *kasB* point mutants (probe *kasB*); right panel, Southern for Δ*kasB* (probe *kasA*). The expected size of each band was: wild-type, 4219 bp; *kasB* point mutants, 7898 bp; Δ*kasB*, 6764 bp. Lane 1, CDC1551 wild-type strain; lane 2, CDC1551 KasB T334A/T336A; lane 3, CDC1551 KasB T334D/T336D; lane 4, CDC1551 Δ*kasB*. **(C)** ***kasB*** **and** ***cmaA2*** **expression levels in the different isogenic mutants.** Analysis of *kasB* and *cmaA2* mRNA levels *of Mtb* strains as determined by quantitative RT-PCR. The mean ± standard deviation of three real-time RT-PCR experiments is shown for each strain. The values were normalized to *sigA* mRNA levels. **(D) KasA/KasB immunoblotting.** Western blotting showing the expression level of KasB in the crude lysates of the parental strain and the various KasB mutant strains. The membrane was probed with rat anti-KasA antibodies which cross-react with KasB and the proteins revealed using secondary antibodies labeled with IRDye infrared dyes. **(E) AccD6 immunoblotting.** Western blot analysis showing the expression level of AccD6 in the crude lysates of the parental strain and the various KasB mutant strains. The membrane was probed with rabbit anti-AccD6 antibodies and the incubated with anti-rabbit antibodies conjugated to alkaline phosphatase.

To study the effect of the two KasB phosphorylation sites in *Mtb*, the Thr334 and Thr336 amino acids were replaced either with phosphomimetic (aspartate) or phosphoablative (alanine) amino acid. Previous studies have shown that acidic residues such as aspartate qualitatively recapitulate the effect of phosphorylation with regard to functional activity [15], [17], [21], [22]. Specialized linkage transduction [23] was used to transfer single point mutant alleles, respectively *kasB* T334D/T336D and *kasB* T334A/T336A in *Mtb* CDC1551 (Table S1 in Text S1, Fig. 2A). These strains contained a *sacB* and *hyg* cassettes inserted between *kasB* and *accD6*. The introduction of the *sacB* and *hyg* cassettes was confirmed by Southern blot (Fig. 2B) and presence of the point mutation(s) was verified by sequencing *kasB*. An additional *kasB* deletion strain in *Mtb* CDC1551 was constructed using the same plasmid (pYUB1471) as the one used for the allelic exchanges.

Quantitative reverse transcription real-time PCR analyses were conducted to measure the *kasB* expression level in the different mutant strains. Expression levels were standardized using the *sigA* internal standard (Fig. 2C). As expected, no specific *kasB* mRNA was produced in the Δ*kasB* mutant. In contrast, *kasB* expression levels were found to be comparable in the parental strain, the double Ala and the double Asp mutants. Similar results were obtained when using either *16S* rRNA or *rrnAP1* as alternative internal control (data not shown). These results were confirmed by Western blot analysis using antibodies raised against KasA, which cross-react with KasB, and revealing comparable levels of KasB expression in the parental strain, the double Ala and the double Asp mutants (Fig. 2D). Moreover, since *kasB* belong to the *fasII* operon and is located upstream of *accD6*, we also checked whether the introduction of the T/A or T/D replacements may affect expression of the downstream *accD6* gene. Immunoblotting using rabbit anti-AccD6 antibodies clearly revealed the presence of similar AccD6 levels in all strains (Fig. 2E). From these data it can be inferred that mutations in *kasB* did not exert a polar effect on AccD6 expression.

Together, these results indicate that introduction of the phosphoablative or phosphomimetic mutations does not affect *kasB* gene expression in *Mtb*, thus allowing analysis and comparison of the phenotypes associated to these mutations.

**Figure 3:**
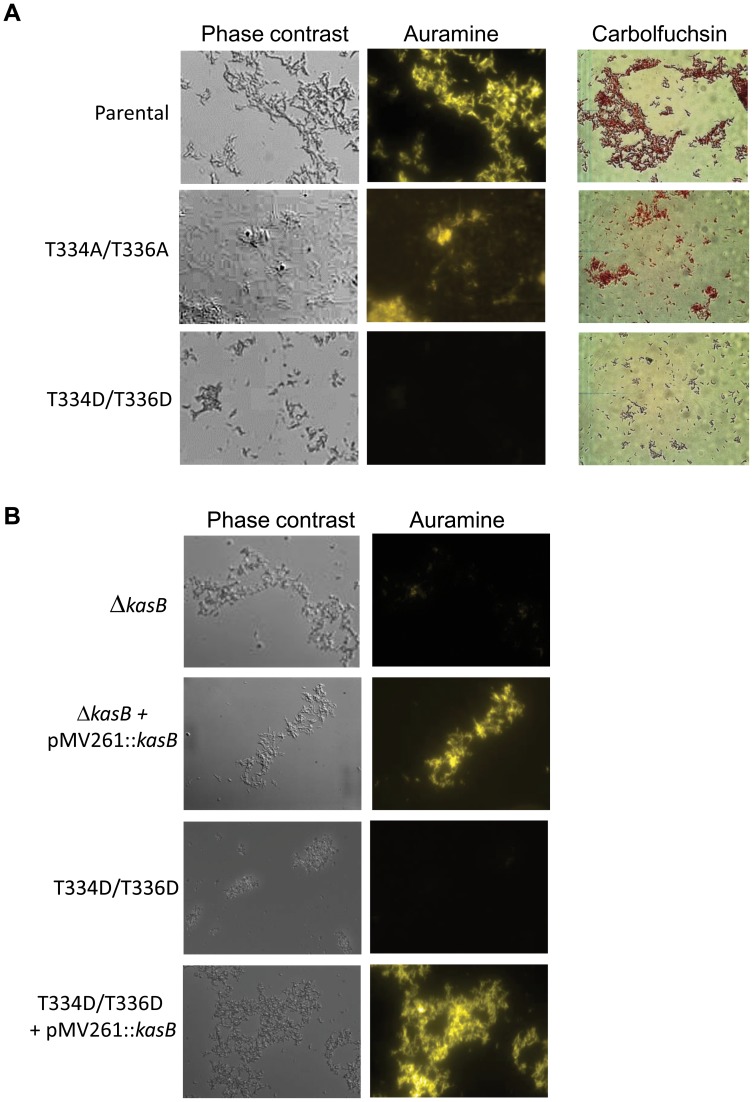
Acid-fast staining of *M. tuberculosis kasB* isogenic mutant strains. (**A**) Cultures were fixed on glass slides and acid-fast staining was performed on the fixed smears using either the BD TB Auramine Kit or the BD Carbolfuchsin kit. The left panels show phase contrast microscopy images of *Mtb* CDC1551 parental strain and the phosphomimetic and phosphoablative *Mtb* isogenic strains. (**B**) Restoration of the acid-fast staining phenotype in the Δ*kasB* and phosphomimetic KasB mutant strains complemented with pMV261::*kasB*. Magnification = 100×.

The first phenotype tested in the KasB mutants was acid-fastness using both the carbolfuchsin and auramine stainings (Fig. 3A). Like the parental strain, the Ala mutant remained acid-fast positive. In sharp contrast, the phosphomimetic T334D/T336D mutant strain behaved like the *kasB* deletion mutant, losing its ability to retain the primary stain following washing with the acid-alcohol decolorizer. The phosphomimetic and deletion strains regained acid-fast staining when a wild-type copy of *kasB* was introduced into these strains on a multicopy plasmid (Fig. 3B). This indicates that phosphorylation on Thr334/Thr336 abrogated the acid-fast property, presumably by negatively regulating the activity of KasB. The results support, for the first time, a control of acid-fastness by STPK-dependent signaling in *Mtb*.

### Phosphorylation of KasB results in shorter mycolic acids

**Figure 4:**
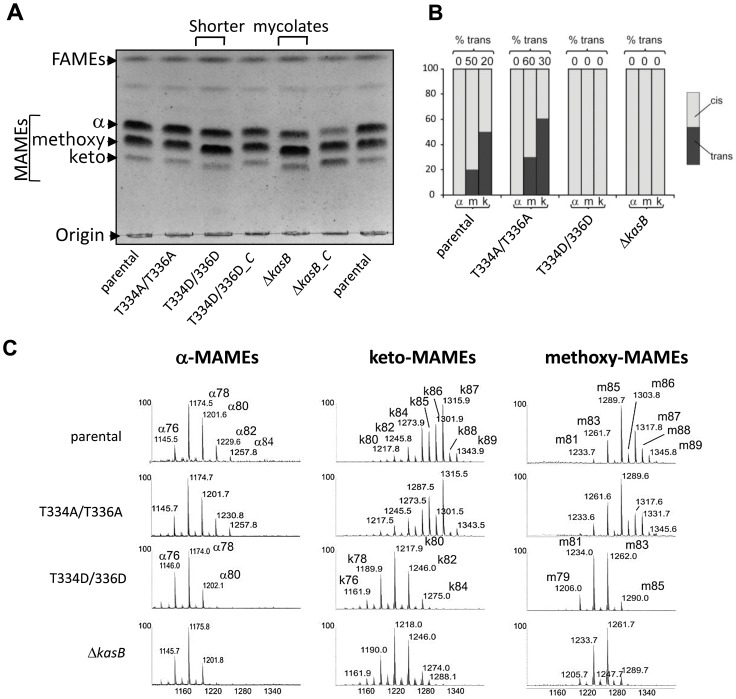
Structural analysis of mycolic acids in the phosphomimetic and phosphoablative *kasB* mutants. (A) Mycolic acid profile of the various KasB mutants and complemented strains. Culture were grown at 37°C, harvested, and FAMEs and MAMEs were extracted and analyzed by one-dimensional TLC using hexane/ethyl acetate (19∶1, v/v; 3 runs). α-, methoxy- and keto-mycolic acids were revealed by spraying the plate with molybdophosphoric acid followed by charring. Mycolates migrating slightly faster in the Asp mutants than in the parental control strain can be observed. “-C” indicates complemented strain (with pMV261::*kasB*). **(B) Relative proportions of** ***cis*** **- and** ***trans*** **-cyclopropanes in mycolates established from ^1^H NMR spectra.** The relative quantification of specific signals associated to *trans*- and *cis*-cyclopropanes revealed that oxygenated mycolates synthesized by the KasB T334D/T336D and Δ*kasB* strains exclusively contain *cis*-cyclopropane rings. The % of *trans*-cyclopropanes for each mycolic acid sub-species and for each strain is indicated. **(C) MALDI-MS analysis.** Mass spectrometry analysis revealed that all three families of mycolates isolated from the KasB T334D/T336D and Δ*kasB* strains display reduced sizes compared to the parental or phosphoablative strains.

The lack of acid-fastness prompted us to investigate the mycolic acid content in the various strains. Since *kasB* deletion results in a strain producing shorter mycolic acids [14], we compared the mycolic acid profiles of the double Ala and double Asp mutants to the parental and Δ*kasB* strains. Mycolic acids were extracted after saponification from the strains grown to stationary phase and analyzed by thin-layer chromatography (TLC) (Fig. 4A). When separated, the mycolates of the parental and phosphoablative strains presented similar mobility shifts, whereas those from the phosphomimetic and the Δ*kasB* strains were found to be slightly retarded. This lower mobility shift has been earlier reported to occur in *M. marinum* and *Mtb kasB* mutants and correlated to reduced carbon chain lengths [14], [24]. The normal mobility shift was restored in the corresponding complemented strains (Fig. 4A).

Individual mycolic acid species from all four strains were then purified from preparative TLC plates, re-extracted and analyzed by proton nuclear magnetic resonance (^1^H NMR) spectroscopy (Fig. 4B and Figure S3 in Text S1) and matrix-assisted laser desorption/ionization-time of flight (MALDI-TOF) mass spectrometry (Fig. 4C). ^1^H NMR analyses revealed that mycolates from the Δ*kasB* and KasB_T334D/T336D strains significantly differed from those of the parental strain. In particular, the relative quantification of signals at −0.33 and 0.55 ppm and signal at 0.45 ppm established that the ratio of *cis*/*trans*-cyclopropanation changed in oxygenated mycolic acids, as reported for the Δ*kasB* mutant [14]. For methoxy-mycolic acids, it decreased from 20% of *trans*-cyclopropanation in parental strain to 0% in the Δ*kasB* and KasB_T334D/T336D strains (Figure S3B in Text S1) and for keto-mycolic acids from 50% to 0% (Figure S3C in Text S1 and Fig. 4B). In contrast, mycolates from the phosphoablative mutant exhibited spectra similar to those of the parental strain (data not shown).

Next, the mycolic acid size distribution was assessed by MALDI-TOF MS, and similarly to the ^1^H NMR analysis, the double Ala mutant was found to produce mycolates identical to those from the parental strain. In contrast, the double Asp mutant, like Δ*kasB*, synthesized shorter α-, methoxy, and keto-mycolic acids (up to C80:2, up to C84:1, and up to C85:1) than the parental strain (up to C84:2, up to C89:1, and up to C89:1), and accumulated a *trans*-unsaturated precursor of both methoxy- and keto-mycolic acids, thus confirming the slightly reduced TLC mobility shift. The average size reduction of oxygenated mycolates was higher than that of α-mycolates because it resulted from both size reduction of aliphatic chain and loss of the -CH3 group of *trans*-cyclopropane groups (Fig. 4C).

### Lack of *trans*-cyclopropanation does not result from altered *cmaA2* expression

NMR analysis indicates the lack of *trans*-cyclopropane rings in oxygenated mycolic acids in the phosphomimetic mutant, which accumulates the corresponding *trans*-double bonds precursors, as found in the Δ*kasB* strain and in agreement with previous work [14]. *Trans*-double bonds have been reported to be the substrates of the *trans*-cyclopropane synthase CmaA2 [25]. We reasoned that lack of *tran*s-cyclopropanation in the phosphomimetic strain mutant may be due to the shortened oxygenated meromycolates that are poor substrates for CmaA2. Alternatively, alteration/lack of *cmaA2* expression may occur in this particular strain. Indeed, multiple direct interactions have been reported to happen between various FASII enzymes in specialized multifunctional complexes [26], [27]. Consequently, phosphorylation of KasB may hinder/prevent association of KasB with other partners, including CmaA2. *cmaA2* expression levels were therefore measured and found to be comparable in all four strains (Fig. 2C), indicating that neither phosphomimetic mutations within KasB nor deletion of *kasB* altered *cmaA2* expression. Similar results were obtained using *sigA*, *16S* rRNA or *rrnAP1* as internal standards (data not shown). This implies that absence of *trans*-cyclopropane rings is likely due to the shortened mycolates that are poor substrate for CmaA2, rather than to a defect in *cmaA2* expression.

### Phosphorylation of KasB leads to increased cell wall permeability

Given the effect of KasB phosphorylation on the meromycolate chain length, we inquired whether shorter mycolic acids would alter drug susceptibility. The KasB strains were tested for growth in the presence of INH, ethionamide (ETH), and rifampicin (RIF) (Table 1). The KasB_T334D/T336D mutant was found to be more susceptible to INH, ETH, and RIF, suggesting that this strain exhibits a cell wall permeability defect.

**Table 1:** MICs of various antitubercular drugs.

MIC (μg/l)	Parental	T334A/T336A	T334D/T336D	Δ*kasB*
**INH**	0.04–0.06	0.04–0.06	0.015–0.03	0.02–0.03
**ETH**	1–2.5	1–2.5	0.25–0.3	1.25–2.5
**RIF**	0.03–0.06	0.016–0.06	0.008–0.015	0.002–0.007

Range of MICs (µg/ml) obtained in two or three independent experiments is shown.

To confirm the permeability defect of this mutant in logarithmic phase, we compared how the phosphomimetic, phosphoablative, deletion and parental strains incorporated two different types of dye: the cyanine dye 3,3′-diethyloxacarbocyanine iodide DiOC_2_, which penetrates all cells, and the SYTOX Red dead cell stain, which is excluded from cells with intact membranes [28], [29]. The highest amount of Sytox Red was found in the KasB phosphomimetic strain (Figure S4 in Text S1), suggesting that this strain had the most permeable membrane. This set of data support the view of a permeability defect in the KasB phosphomimetic strain.

### Reduced pathogenicity and mortality of the KasB phosphomimetic strain

**Figure 5:**
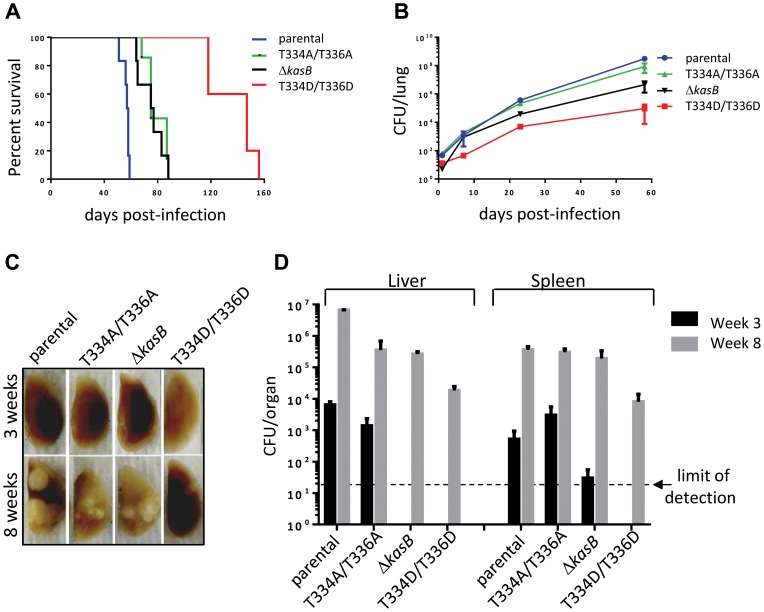
Infection of immunocompromised SCID mice with *M. tuberculosis kasB* isogenic mutants. (A) Survival curves in infected SCID mice. Low-dose aerosol infection of SCID mice (as in **B**) was performed with the following *Mtb* strains: parental, KasB_T334A/T336A, KasB_T334D/T336D and Δ*kasB*. **(B) Growth of** ***Mtb kasB*** **strains in the lungs of SCID mice.** At 1, 7, 21 and 56 days post-infection, one lung from each infected SCID mouse was harvested, homogenized and serial dilutions were plated on Middlebrook 7H10 supplemented with 10% OADC and 0.2% glycerol. **(C) Pathology of lungs from infected SCID mice.** One lung from each infected SCID mouse was harvested and fixed in 10% paraformaldehyde for a month prior to photography. **(D) CFU plots in the liver and spleen three weeks (black bars) and eight weeks (grey bars) post-infection.**

Growth curves of *Mtb* bearing either the *kasB_T334A/T336A* or the *kasB_T334D/T336D* allele were also similar, indicating that the phosphomimetic or phosphoablative mutations did not impact *in vitro* growth (data not shown). To address the physiological relevance of KasB phosphorylation with respect to *Mtb* virulence and physiopathology, low-dose aerosol infection of mice were performed with the parental, double Ala, double Asp and Δ*kasB* isogenic *Mtb* strains. Previous work showed that whereas CDC1551 Δ*kasB* was severely attenuated and failed to cause active infection in immunocompetent mice, it caused mortality in immunodeficient SCID mice [14]. Therefore, *in vivo* growth of the different KasB mutants was tested in both SCID mice and immunocompetent C57BL/6 mice. Although all the strains grew in the lung of SCID mice, the double Asp was found to be much more attenuated than the other KasB variants. Indeed, the phosphomimetic mutant strain was strikingly less virulent relative to the other strains following aerogenic challenge, as assessed by the mean survival time of SCID mice infected with these strains (57.5, 75, 76 and 147 days post-infection for parental, KasB_T334A/T336A, Δ*kasB* and KasB_T334D/T336D, respectively). Mice infected with the double Asp mutant survived on average 90 days longer than the mice infected with the parental strain, while the mice infected with the double Ala or Δ*kasB* strain survived only 20 days longer than the mice infected with the control strain (Fig. 5A). In agreement with the survival data, quantification of tissue bacterial burden revealed a severe growth defect (3–4 Logs) of the KasB phosphomimetic strain (Fig. 5B). Manifestation of this hypovirulent phenotype is apparent as early as one week post-infection with the lung bacterial burden of mice infected with *Mtb* KasB_T334D/T336D about 30-fold lower than the parental strain-infected mice. The bacterial burden of the *Mtb* KasB_T334A/T336A strain was comparable to that of the parental strain. Consistent with previously published data [14], the Δ*kasB s*train exhibited a growth defect, albeit less pronounced than the *Mtb* KasB_T334D/T336D strain (Fig. 5B). Extensive granulomatous inflammation was visible in the lungs of SCID mice infected with the parental strain, and to a lesser extent in mice infected with the KasB_T334A/T336A and the Δ*kasB s*trains but not in those infected with the KasB_T334D/T336D (Fig. 5C). Monitoring of colony-forming units (CFU) in spleen and liver at three and eight weeks post-infection revealed that the KasB_T334D/T336D mutant was also severely attenuated for growth in these organs (Fig. 5D). The restricted bacterial loads in both organs increase by three orders of magnitude after eight weeks of infection. In contrast, replication of the KasB_T334A/T336A strain was comparable to that of the parental strain in both organs, although a 1-log growth defect was observed in the liver eight weeks after infection, whereas replication of the Δ*kasB* mutant was also severely impeded in these two organs.

**Figure 6:**
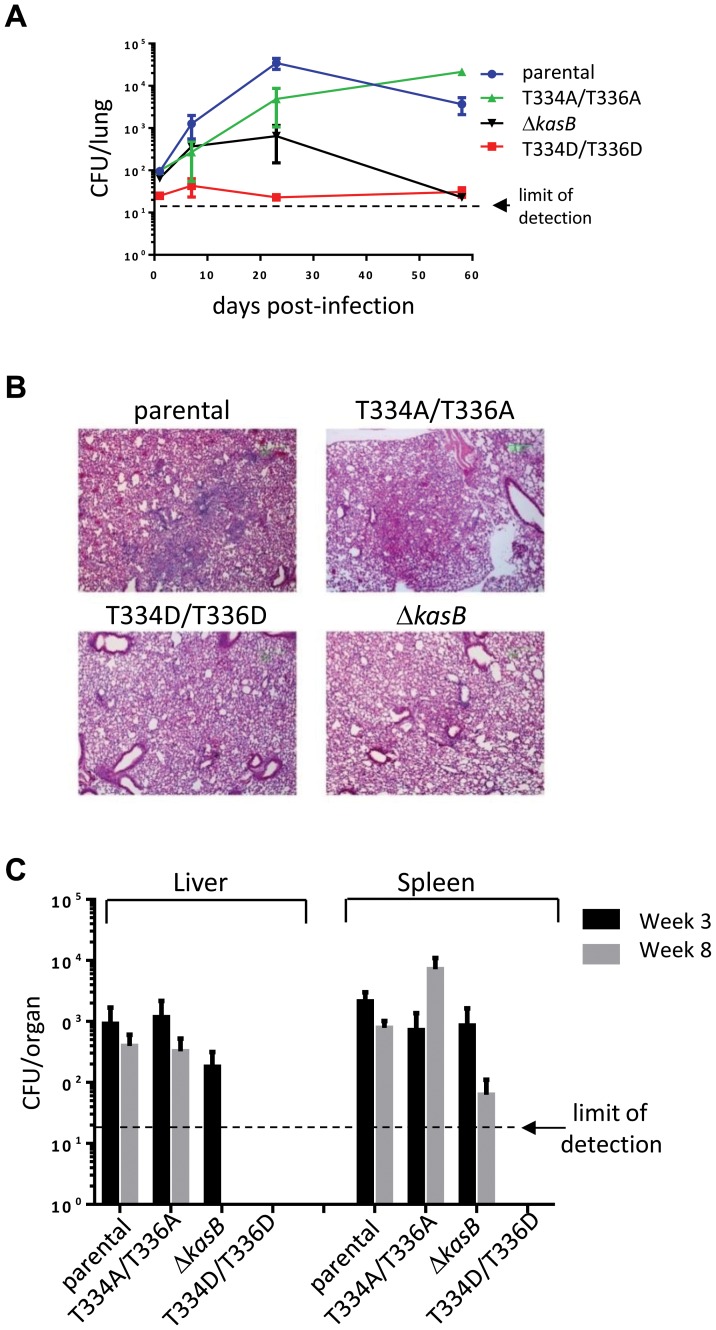
Infection of immunocompetent C57Bl/6 mice with *M. tuberculosis kasB* isogenic strains. (A) Growth of *Mtb kasB* strains in the lungs. Low-dose aerosol infection was performed with the following *Mtb* strains: parental, KasB_T334A/T336A, KasB_T334D/T336D, and Δ*kasB*. Lungs from infected mice were harvested at 1, 7, 21 and 57 days post-infection, homogenized and serial dilutions were plated onto Middlebrook 7H10 plates supplemented with 10% OADC and 0.2% glycerol. **(B) Pathology slides of lungs from infected mice at 21 days post-infection.** Lung tissue sections from mice infected with the *Mtb* CDC1551 parental, KasB_T334A/T336A, KasB_T334D/T336D, and Δ*kasB* were stained with hematoxylin/eosin and observed. Magnification = ×20. **(C) CFU plots in the liver and spleen three weeks (black bars) and eight weeks (grey bars) post-infection.**

Next, immunocompetent C57BL/6 mice were infected via aerosol and assessed for both survival and bacterial replication. Monitoring of CFU in the lungs at different time points after infection indicated that both the parental and double Ala mutant grew for the first three weeks following a similar kinetic (Fig. 6A). Consistent with previous observations, the Δ*kasB* was severely attenuated for growth in mice [14]. In contrast to the Δ*kasB*, the KasB phosphomimetic mutant failed to replicate, even after the first three weeks of infection and was never found in the lungs of C57BL/6 in two independent aerosol infection experiments. Histological examination of stained lung sections from infected mice revealed multifocal, moderate infiltration after 21 days of infection with the parental strain (Fig. 6B) but not with the KasB_T334A/T336A, Δ*kasB* or KasB_T334D/T336D mutant strains, which may be attributed to the lower bacterial loads of these strains at this particular time point. Bacterial loads in the liver and spleen at different time points after infection indicated that the KasB phosphomimetic mutant was extremely attenuated, since CFU could not be obtained at either 3 or 8 weeks post-infection (Fig. 6C). Replication of the Δ*kasB* mutant was also severely impeded in these two organs.

Together, this suggests that KasB phosphorylation regulates the growth of *Mtb* in both immunocompromised and immunocompetent mice. In the presence of phosphorylated KasB, the tubercle bacillus fails to establish a chronic persistent infection, and exhibits a severely attenuated phenotype.

### KasB phosphorylation reduces the uptake of *M. tuberculosis* by macrophages

**Figure 7:**
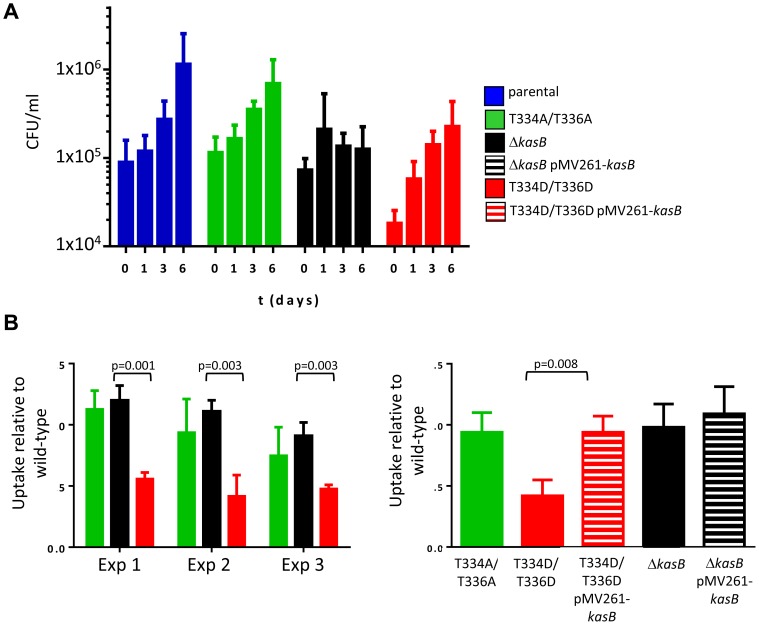
Infection of C57BL/6 bone marrow-derived macrophages with *M. tuberculosis kasB* variants. (A) Growth of the KasB mutants and parental strain in BMDM. An MOI of 1 was used to infect the BMDM with the strains. Macrophages were lysed after 0, 1, 3 and 6 days post-infection and viable bacteria were counted by plating dilutions of the lysates on agar plates. Three independent experiments were performed and each experiment was done in triplicate. **(B) Uptake of KasB strains by BMDM.** The ratio between the bacterial titer after a 4 h infection and the inoculum titer is shown. A T-test (two-tailed distribution, unequal variance) was performed and showed a statistical difference between the uptake of the KasB phosphomimetic strain compared to deletion strain (left panel). Complementation with a functional KasB restores the uptake of the phosphomimetic mutant (right panel). Blue bar, parental strain; green bar, KasB_T334A/T336A; black bar Δ*kasB*, black bar with white stripes, Δ*kasB* pMV261-*kasB*; red bar, KasB_T334D/T336D; and red bar with white stripes, KasB_T334D/T336D pMV261-*kasB*.

The difference in virulence between the Δ*kasB* and the phosphomimetic strains led us to investigate how these two strains infect and survive in macrophages, the primary cellular targets of *Mtb*. C57BL/6 bone marrow-derived macrophages (BMDM) were infected with the various *Mtb* KasB variants, lysed at 0, 1, 3 and 6 days post-infection, and the numbers of viable bacteria were counted (Fig. 7A). Both the parental and the KasB_T334A/T336A mutant grew similarly whereas the Δ*kasB* mutant, as expected, was attenuated for growth in macrophages, with day 6 CFU counts similar to day 0 numbers, versus an approximately one log increase for the parental strain. In contrast, the KasB phosphomimetic strain had no significant growth defect in macrophages but exhibited a marked impairment in macrophage uptake (Fig. 7A, day 0 time point). This effect was further investigated by measuring internalization of the various strains in BMDM. In three independent experiments, the uptake of the KasB phosphomimetic strain was significantly lower than the one of the parental strain by around 50% (Fig. 7B, left panel), a defect that was reversed when the phosphomimetic strain was complemented with a wild-type *kasB* on a multicopy plasmid (Fig. 7B, right panel). To confirm that these macrophages phenotypes were not specific to C57BL/6 BMDM, the growth of the KasB strains was also tested in the human acute monocytic leukemia cell line THP-1. In THP-1 macrophages, the phosphomimetic KasB mutant had also no growth failure and did present an uptake defect similar to the one observed in BMDM (Figure S5 in Text S1). Taken together, this set of data suggests that phosphorylation of KasB regulates the early interaction event between *Mtb* and macrophages.

### Increased polyacyled trehalose levels in the KasB phosphomimetic strain

The distinct *in vivo* phenotypes between the KasB phosphomimetic mutant and the Δ*kasB* mutant may reflect different physiological/metabolic status. To identify transcriptional differences that may be relevant to the differential phenotypes of these strains, a whole-genome transcriptional analysis was performed (Figure S6 in Text S1). Several genes were commonly up- or down-regulated two-fold or more in the KasB mutant strains. Most up-regulated genes were genes participating in cell wall, cell processes and lipid metabolism, whilst the majority of the down-regulated genes were associated with intermediary metabolism and respiration. Interestingly, a few genes were uniquely up-regulated in the KasB phosphomimetic strain such as the esterase/lipase *lipF*, proposed to play an important role in *Mtb* pathogenesis [30], and five genes involved in lipid or drug transport (MmpL4, MmpL5, MmpL10, MmpS4, and Rv1258c) [31], [32], as well as five other genes involved in lipid metabolism (Pks3, Pks16, PapA1, PapA3, and FadD21). The genes specifically down-regulated in the KasB phosphomimetic mutant encoded: NrdF1, an enzyme involved in DNA replication; two oxidoreductases (Rv3741c, Rv3742c) which are in an operon with a triacylglycerol synthase; two enzymes (Rv3084, Rv3085) part of the *mymA* operon which might be implicated in the modification, activation and transfer of fatty acids to the cell envelope [33]. Strikingly, most of the biosynthetic gene cluster (*pks3/4, papA3, mmpL10, fadD21*) required for the synthesis of polyacylated trehalose (PAT) [34] was specifically up-regulated in the KasB phosphomimetic mutant suggesting that PAT biosynthesis in this mutant might be altered.

**Figure 8:**
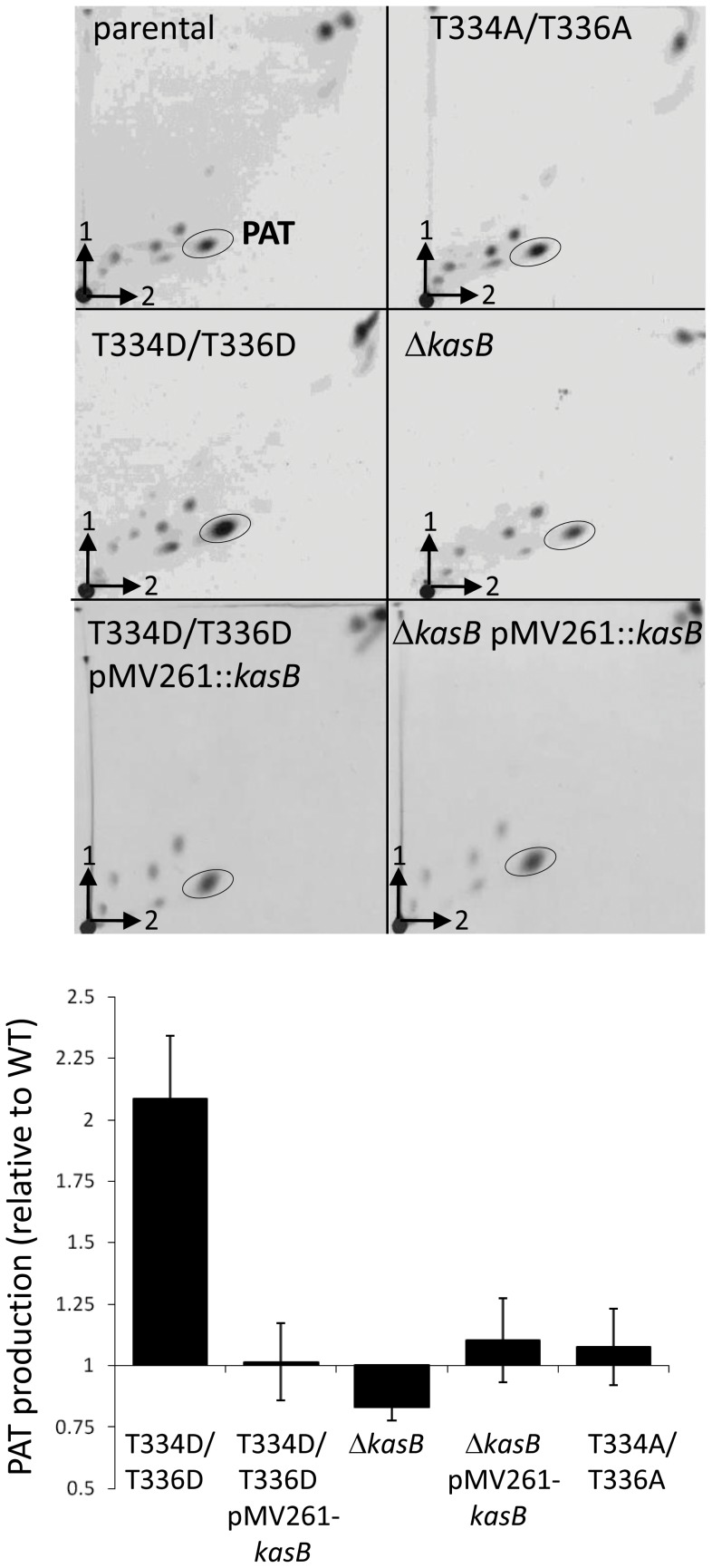
Expression of PAT in the *M. tuberculosis kasB* variants. Left panel: autoradiographs of thin layer chromatograms of apolar lipids derived from [1-^14^C]-propionate labeling in the various *Mtb* KasB strains and complemented strains carrying pMV261-*kasB*. Total lipids (6,000 counts) were loaded on TLC plates and developed thrice in petroleum ether/acetone (92∶8, v/v) in the first direction and once in toluene/acetone (95∶5, v/v) in the second direction. Right panel: quantification of the PAT production (corresponding to the circled spots in left panel). Results are expressed in fold increase relative to the parental strain. Results are representative of two independent experiments.

Metabolic labeling with ^14^C-propionate and subsequent lipid analysis of parental, phosphoablative, phosphomimetic and deletion strains showed that the phosphomimetic KasB mutant produced more PAT than the three other strains (Fig. 8). Quantification of the TLC spots revealed a reproducible two-fold increase in PAT production in the KasB_334D/336D mutant compared to the three other strains and to its complemented strain, suggesting that phosphorylation of KasB positively affects PAT synthesis.

## Discussion

**Figure 9:**
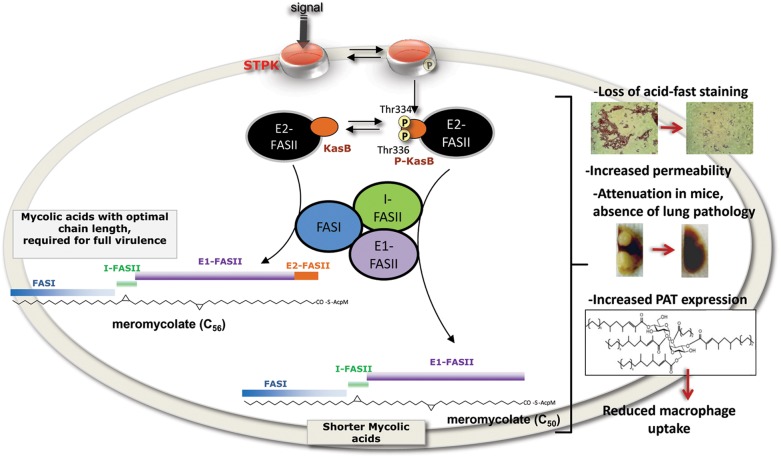
Representation of the *in vivo* consequences of STPK-dependent phosphorylation of KasB. Changes in cell wall and mycolic acid composition to various environmental stimuli are central to *Mtb* adaptation during infection. In response to external cues, STPKs undergo autophosphorylation, which in turn induces phosphorylation of KasB on Thr334 and Thr336. This presumably results in inactivation of KasB activity, thus directly affecting the activity of the elongation 2 FASII complex (E2-FASII) catalyzing the addition of the last carbon atoms required for full-length meromycolic acids. This leads to the production of shorter mycolic acids which is associated to dramatic phenotype changes, such as loss of acid-fastness, decreased cell wall permeability, severe attenuation in infected mice and defect in macrophage colonization.

Recent studies have provided clear insights into the vast range of pathways regulated by STPK in *Mtb*. These include multiple metabolic processes, transport of cell wall components as well as cell division or virulence functions [5], [6], [7]. Several STPK, such as PknH or PknG, have been reported to play a crucial role in modulating *Mtb* virulence [6], [35], [36], [37], [38]. However, little is known regarding the specific substrates contributing to mycobacterial virulence and regulated by these kinases. Here, we report the critical role of STPK-dependent phosphorylation of KasB, which is directly linked to *Mtb* virulence. Through the design of a KasB phosphomimetic *Mtb* mutant, we demonstrate that, *in vivo*, the replacement of the two Thr by Asp residues was characterized by highly pronounced phenotypes corresponding to i) loss of acid-fastness, ii) production of shorter mycolic acids with defects in *trans*-cyclopropanation, iii) defect in macrophage invasion, iv) incapacity to grow and establish a persistent infection in both immune-compromised and immune-deficient mice, and v) absence of pathology in infected animals (Fig. 9). The long-term persistence of the KasB phosphomimetic strain without causing disease or mortality makes it an attractive model for studying latent *Mtb* infections and suggests that this attenuated strain may represent a valuable vaccine candidate against TB.

By analogy with a Δ*kasB* mutant, these phenotypes strongly support the view that phosphorylation has a detrimental effect on KasB activity. This is also emphasized by the modelling studies highlighting the strategic localization of the two phosphosites in the vicinity of the catalytic triad. That phosphorylation of these residues is likely to perturb the condensing activity of KasB contrasts with an earlier study proposing that phosphorylation of KasA and KasB reduces and increases their condensing activity *in vitro*, respectively [8]. The discrepancy between these two studies could be explained by several reasons. First, in the *in vitro* study, KasB activity was assessed using C_16_-AcpM, which represents a preferred substrate for KasA, but not for KasB [39] which catalyzes the last step in the elongation cycle [14], therefore acting on very long fatty acyl chains, such as C_48_-C_52_-AcpM substrate. Second, to conduct these studies, preparation of purified KasA or KasB from recombinant BCG were used, which contain large proportions of non-phosphorylated proteins, as usually within bacteria only a small proportion of proteins gets phosphorylated. A heterogeneous preparation containing a mixture of phosphorylated and non-phosphorylated isoforms may affect the overall activity of KasB. Finally, several studies have shown that most FASII components interact together [26], [27], [40] and a “mycolome” concept has recently emerged from work demonstrating that FASII system of *Mtb* is organized in specialized interconnected complexes composed of the condensing enzymes, dehydratase heterodimers and the methyltransferases [26], [27], [40]. In this model of interactome, three types of FASII specialized complexes are interconnected together: i) the initiation FASII is formed by a core consisting of the reductases, FabD and FabH, linking FASI and FASII together; ii) two elongation FASII complexes consisting of a core and either KasA (E1-FASII) or KasB (E2-FASII) which are capable of elongating acyl-AcpM to produce full-length meromycolyl-AcpM (Fig. 9); and iii) the termination FASII involving Pks13 which condenses the α branch with the meromycolic branch. One may therefore hypothesize that phosphorylation of KasB may also alter/disrupt heterotypic interactions with other FASII partners of E2-FASII which may in turn affect the activation of E2-FASII, leading to shortened mycolates. This would be missed in *in vitro* assays using single purified proteins.

To address the possibility that phosphorylation of KasB could also affect the localization of the protein, recombinant strains of *M. smegmatis* and *M. bovis* BCG expressing green fluorescent protein (GFP)-tagged KasB variants proteins were produced, comprising wild-type KasB, a phosphoablative (T334A/T336A) and a phosphomimetic (T334D/T336D) KasB isoform. We found that all KasB/eGFP fusion proteins localized in the mycobacterial cell wall, mostly on the bacterial poles (Figure S7 in Text S1). These findings strongly suggest that mycolic acid biosynthesis takes place during mycobacterial division at the bacterial poles and indicate that phosphorylation of KasB does not affect its localization in mycobacteria. Therefore, one can hypothesize that the primary consequence of KasB phosphorylation *in vivo*, is very likely to result in alteration of its enzymatic activity.

A recent study revealed that there were no detectable differences in the thickness of the cell envelope among the wild-type *Mtb* and Δ*kasB* mutant as determined by conventional transmission electron microscopy [41]. However, cryo-electron microscopy demonstrated that the region between the inner and outer membranes of the mutant strain, mainly composed of mycolic acids, showed a significant decrease in electron density as compared to the wild-type strain, suggesting that the altered mycolic acid pattern in the Δ*kasB* mutant may have affected the packing of the lipid-rich layer of the envelope [41]. One may hypothesize that the attenuated pathogenicity of the KasB phosphomimetic strain results in the increased permeability of its cell envelope due to the reduction in the number of tight bundles of mycolic acids, which facilitates the direct attack of effector molecules from the host cells. This altered mycolic acid packing and/or reduced chain length may also be responsible for the reduced capture of the dye, leading to loss of acid-fastness.

Previously, we have demonstrated that the mycolic acid cyclopropane synthase PcaA, which introduces *cis*-cyclopropane rings at the proximal position in α-mycolic acids, was phosphorylated by STPK [17]. As for KasB, phosphorylation of PcaA was associated with a significant decrease in the enzymatic activity. A PcaA phosphomimetic (T168D/T183D) strain, like the Δ*pcaA* mutant, exhibited reduced survival in human macrophages and was unable to prevent phagosome maturation, compared to the wild-type strain. This added new insights into the importance of mycolic acid cyclopropane rings in the phagosome maturation block and provided the first evidence of a Ser/Thr kinase-dependent mechanism for modulating mycolic acid composition and phagosome maturation block [17]. The present study extends these results by adding KasB in the growing list of virulence factors associated with mycolic acid metabolism, whose activities are directly regulated by phosphorylation. However, in contrast to PcaA, phosphorylation of KasB significantly altered colonization of macrophages and, unexpectedly, this phenotype was not shared by the Δ*kasB* strain. These differences were reflected in the different transcriptional profiles between the two strains. Among these transcriptional differences, specific up-regulation of the PAT biosynthetic gene cluster occurred in the phosphomimetic strain and correlated with increased production of PAT. Earlier studies demonstrated that PAT deficiency affects the surface global composition of the mycobacterial cell envelope, improving the efficiency with which *Mtb* binds to and enters phagocytic host cells [42], implying that PAT production affects early interaction between *Mtb* and macrophages. From these results, one can propose that the increased PAT production in the KasB phosphomimetic strain could be, at least partially, responsible for the decreased uptake by macrophages, which in turn may also explain the attenuation of the phosphomimetic mutant in mice. As shown in Figure S8 in Text S1, the phosphomimetic strain also produced PDIM, thus excluding the possibility that the attenuated phenotype of the phosphomimetic strain could be a consequence of the loss of PDIM. However, as suggested by the microarray data, multiple genes belonging to different classes such as adaptation, cell wall processes, intermediary metabolism, and respiration or regulatory proteins were found to be differentially regulated between the KasB phosphomimetic and knock-out strains. They may also, in addition to the PAT biosynthetic genes, participate in the phenotypic differences between the two strains.

Analysis of the phosphorylation status of KasB in BCG using anti-phosphothreonine antibodies indicated that phosphorylation was more prominent in stationary cultures than in replicating cultures. These finding are reminiscent of those reporting that phosphorylation of the FASII HadAB and HadBC complexes is growth phase-dependent and that phosphorylation occurred at higher levels in non-replicating bacteria [12], suggesting that phosphorylation is a mechanism by which mycobacteria might tightly control mycolic acid biosynthesis under non-replicating conditions. *Mtb* bacilli have two signature characteristics: acid-fast staining and the ability to cause long-term latent infections in humans. Acid-fast staining, such as Ziehl-Neelsen (ZN) staining, remains the cornerstone of diagnosis of TB, particularly in poor countries where the infection is highly prevalent [43]. Dormant bacilli have distinct structural alterations in the cell wall and are ZN-negative [44]. It is noteworthy that in a high percentage of patients exhibiting TB symptoms, analysis of patient's tissue samples may be positive for culture of mycobacteria and PCR analysis but negative for ZN staining. However, the reason(s) for the loss of acid-fastness during dormancy remain(s) unknown. A connection between loss of acid-fast staining and latent infection had been reported for a *kasB* deletion mutant [14] suggesting that regulation of KasB activity may cause these linked phenotypes. The present work provides the first evidence that phosphorylation of KasB correlates the loss of acid-fast staining and a loss of virulence allowing us to hypothesize that *Mtb* regulates these two related phenotypes through a signal transduction pathway. Further work will need to be done to elucidate these signals and determine which specific kinases regulate them *in vivo*. This knowledge might lead to better understanding of the molecular signals that trigger reactivation and TB disease. Although we show here that phosphorylation of KasB was more pronounced in stationary phase, additional studies are required to demonstrate whether this also happens in persistent mycobacteria and if it would result in the loss of acid-fast staining. This would open the way to improved methods for the diagnosis of latent TB infections.

## Materials and Methods

### Ethics statement

All animal experiments and protocols described in the present study were reviewed and approved by the Animal Use and Care Committee of the Albert Einstein College of Medicine (Bronx, NY) complying with NIH guidelines under the Animal Study Protocol 20120114.

### Bacterial strains, plasmids, phage and growth conditions

Strains used for cloning and expression (Table S2 in Text S1) of recombinant proteins were *E. coli* TOP10 (Invitrogen) and BL21(DE3)pLysS (Novagen) or BL21(DE3)Star (Novagen) grown in LB medium at 37°C. Media were supplemented with ampicillin (100 µg ml^−1^) or kanamycin (25 µg ml^−1^), as required. Mycobacterial strains used (Tables S1 and S2) were usually grown on Middlebrook 7H10 agar plates with OADC (oleic acid, albumine, dextrose, catalase) enrichment (Difco). Liquid cultures were obtained by growing mycobacteria either in Sauton's medium or in Middlebrook 7H9 (Difco) supplemented with 10% OADC enrichment, 0.2% (v/v) glycerol, and 0.05% (v/v) tyloxapol (Sigma) supplemented with either kanamycin (25 µg ml^−1^) or hygromycin (75 µg ml^−1^) when required. The multicopy expression plasmid pVV16 was reported earlier [45]. All plasmids used in this study are listed in Table S2 in Text S1 and the shuttle phasmid phAE159 was described previously [18], [46], [47].

### Cloning, expression and purification of recombinant KasB-derived mutant proteins

The *kasB* gene was cloned into pCR-bluntII-TOPO using primers NtermKasB and CtermKasB (Table S3 in Text S1) to yield pCR-bluntII-TOPO::*kasB*. Site-directed mutagenesis was performed directly on pCR-bluntII-TOPO_*kasB* using inverse-PCR amplification with self-complementary primers (Table S3 in Text S1) carrying the desired mutation. The modified genes were subcloned in pETPhos using NdeI and NheI restriction sites, generating pETPhos_*kasB*_WT, pETPhos-*kasB*_T334A/T336A and pETPhos_*kasB*_T334D/T336D (Table S2 in Text S1). All constructs were verified by DNA sequencing. Recombinant KasB proteins were overexpressed in *E. coli* BL21(DE3)Star (Novagen) and purified as described previously [9]. Fractions containing pure KasB proteins were pooled, dialyzed when required and stored at −20°C until further use. The *kasB* gene was further subcloned from pETPhos_*kasB*_WT by *Nco*I/*Hind*III digest and ligated into pCDFDuet_1 vector already containing the PknF kinase domain [18], thus yielding pETDuet_*kasB* which allows co-expression of both PknF and KasB (Table S2 in Text S1).

A Δ*kasB* BCG mutant was transformed with either pVV16_*kasB*_WT [8] or pVV16_kasB_T334A/T336A which was derived from pVV16_*kasB*_WT by site-directed mutagenesis using inverse-PCR amplification with self-complementary primers carrying the desired mutation (Table S3 in Text S1). Transformants were selected on Middlebrook 7H10 supplemented with OADC enrichment, 50 µg ml^−1^ hygromycin and 25 µg ml^−1^ kanamycin and grown in Sauton's broth containing the same antibiotics. Exponential and stationary phase cultures were harvested, lyzed using a French Pressure Cell and purification of soluble KasB and KasB_T334A/T336A proteins was performed on Ni-NTA agarose beads as described earlier [8].

### Construction of transducing mycobacteriophages carrying *kasB* mutations

Mycobacteriophages used for transduction were prepared as described previously [47]. Briefly, *kasB* was PCR-amplified from the plasmids pETPhos_*kasB* carrying either the T334A/T336A or the T334D/T336D double mutations using the primers LL2 and LR1 (Table S3 in Text S1). *accD6* was PCR-amplified using the primers RL and RR (Table S3 in Text S1). The *kasB* and *accD6* PCR fragments were digested with AlwN1 and Van9I1, respectively and ligated with the 1.6 kb and 3.6 kb fragments of Van9I1-digested pYUB1471 (Table S2 in Text S1). The resulting plasmids were digested with PacI and ligated with the Pac1-digested shuttle phasmid phAE159. After packaging *in vitro*, the resulting phasmids were electroporated into *M. smegmatis* and the phages were amplified to obtain high-titer phage lysates.

### Specialized transduction experiment

*Mtb* (50 ml) was grown to log phase, washed twice with mycobacteriophage (MP) buffer (50 mM Tris, 150 mM NaCl, 10 mM MgCl_2_, 2 mM CaCl_2_; 50 ml) and resuspended in 5 ml of MP buffer. For each transduction experiment, 0.5 ml of cell suspension was mixed with 0.5 ml of high-titer phage lysate. The suspension was incubated at 37°C for 4 h without shaking, spun down, resuspended in 0.2 ml of 7H9 media (see above) and plated on 7H10 plates supplemented with hygromycin. Plates were incubated at 37°C for 4–5 weeks and transductants were picked and patched onto two hygromycin-containing plates. Colonies from one Hyg plate were used for DNA isolation using InstaGene Matrix (BioRad). PCR was performed using 5 µl of DNA for a 50 µl PCR reaction with the primers kasB_F and kasB_R (Table S3 in Text S1) and the resulting products were sequenced to check for the presence of the desired mutations. Southern blotting was done on genomic DNA isolated from Hyg-resistant transductants digested with *Bgl*II and probed with the *kasB* or *kasA* gene to confirm the allelic exchange and *kasB* deletion, respectively.

### Acid-fast staining

The *kasB* mutants were grown to log phase and 10 µl of culture were spread onto a glass slide. The slides were heated at 100°C for 2 min, dipped into 10% formalin for 30 min, dried and stained using the TB Fluorescent Stain Kit M (BD, Auramine staining) or the TB Stain Kit K (BD, Carbolfuchsin staining).

### Mouse experiments

SCID mice and C57BL/6 mice (Jackson Laboratories) were infected via the aerosol route using a 2×10^6^ CFU/ml mycobacterial suspension in PBS containing 0.05% tyloxapol and 0.04% antifoam. Five mice from each group were sacrificed at day 1, 7, 21 and 56 to determine the bacterial burden in the lung, spleen, and liver (one aerosol experiment was carried on for 119 days). Six mice per group were kept for survival experiments. All mice infected with *Mtb* were maintained under appropriate conditions in an animal biosafety level 3 laboratory.

### Growth in primary macrophages

C57BL/6 mice were used to obtain bone marrow-derived macrophages (BMDM). Isolated femurs were flushed with Dulbecco modified Eagle medium (DMEM; Gibco) supplemented with 10% heat-inactivated fetal bovine serum (FBS), 2 mM L-glutamine, and 1× non-essential amino acids (complete DMEM). The cells were cultured for 7 days in complete DMEM containing M-CSF (ebioscience) at 30–50 ng/ml, and then seeded into 24-well plates (∼4×10^5^ cells/well) or 48-well plates (∼2×10^5^ cells/well) as described previously [48]. The cells were allowed to adhere overnight prior to infection. The strains were grown as described above, washed and resuspended in DMEM supplemented with 10% FBS and diluted in this medium to achieve the appropriate titer. The bacteria were added to the wells at an approximate multiplicity of infection (MOI) of 1. Following 4 hrs incubation at 37°C to permit bacterial uptake, macrophage monolayers were washed twice with PBS to remove extracellular bacteria, following which wells were replenished with complete DMEM containing M-CSF at 10–50 ng/ml. At various times after infection, the medium in each well was removed to a tube containing sufficient SDS to give a final concentration of 0.025%; the cell monolayers were lysed with 0.025% SDS and combined with the medium. Lysates were diluted in PBS and plated onto Middlebrook 7H10 (see above) for determination of bacterial numbers.

### RNA extraction for qRT-PCR

*Mtb* strains were cultured to an OD_600_ of 0.5 in 7H9 broth supplemented with OADC, 0.2% glycerol and 2.5 ml 20% Tween 80. Cells were pelleted at 4500 rpm in 15 ml falcon tubes for 15 min, decanted, resuspended in 1 ml Trizol reagent (Invitrogen), and then transferred into new 2 ml screw cap microcentrifuge tubes containing 0.5 ml of zirconia-silica beads (diameter, 0.1 mm). Bacteria were disrupted in a Bead Beater (FastPrep Cell Disrupter, FP120) using two 45 second pulses at maximum speed of 6.5 m/sec, incubated on ice for 5 min, then 250 µl of chloroform was added, following by vigorous mixing for 15 s and then a 2–3 min room temperature incubation. Samples were then centrifuged at 12, 000 *g* for 5 min and the upper clear phase transferred carefully to a new tube and mixed with an equal volume of 70% ethanol, applied to an RNeasy mini column (QIAGEN) and processed according to the manufacturer's recommendations.

### Reverse transcription real time PCR

Five mRNA targets (*kasB*, *cma2*, *sigA*, *16S* and *rrnAP1*) were reverse-transcribed, using a QuantiFast Multiplex RT-PCR kit (QIAGEN) according to the manufacturer's recommendations using a primer specific for each target gene. The conditions for Reverse Transcription PCR were 50°C for 50 min, 95°C 2 min. The amount of cDNA produced was quantified by real time PCR with the corresponding molecular beacon. All primer and molecular beacon sequences are listed in Table S4 in Text S1. The 10 µl PCR reaction mixture consisted of 1× PCR buffer, 250 µM dNTPs, 4 mM MgCl_2_, 0.5 µM each primer, 5 ng/µl molecular beacon and 0.03 U/µl Jumpstart Taq polymerase (Sigma-Aldrich). In order to normalize the individual reactions 6-carboxy-x-rhodamine (ROX) was always included as passive reference dye. PCRs were performed in 384-well microtiter plates in an ABI 7900 Prism (Applied Biosystems, Foster City, CA) according to the following parameters: initial denaturation at 95°C for 1 min, followed by 50 cycles of denaturation at 95°C for 30 seconds, annealing at 58°C for 30 seconds, and extension at 72°C for 15 seconds. PCR conditions were identical for all assays. The fluorescence was recorded during the annealing step of the assay. The quantity of specific target DNA was determined from the threshold cycle (CT) value with reference to a standard curve of genomic DNA. The copy numbers of target standards used ranged from 1 to 10E^6^ genomic copies per reaction (i.e. 10 fg to 10 ng DNA from CDC1551 strains). The lower limit of detection for each of the five assays was 10 fg which is equivalent to 1–5 copies of cDNA. RT reactions were performed in triplicate. The data for *kasB* and *cmaA2* was normalized against 3 different control genes viz. *sigA*, *rrnAP1* and *16S* rRNA [49], [50], [51]. The mean of each triplicate was used in calculations.

### Microarrays

Triplicate samples were prepared by harvesting growing cultures of *Mtb* CDC1551 KasB strains (OD_600 nm_≈1). Extraction of RNA, preparation of cDNA, and microarray analysis were performed as described previously [52]. The array data have been deposited in the Gene Expression Omnibus at NCBI with accession number GSE47640.

### *In vitro* kinase assay

*In vitro* phosphorylation was performed as described [53] with 4 µg of KasB in 20 µl of buffer P (25 mMTris-HCl, pH 7.0; 1 mM DTT; 5 mM MgCl_2_; 1 mM EDTA) with 200 µCi ml^−1^ [γ-^33^P]ATP corresponding to 65 nM (PerkinElmer, 3000 Ci.mmol^−1^), and 0.2 to 1.0 µg of PknF kinase in order to obtain its optimal autophosphorylation activity for 30 min at 37°C. Cloning, expression and purification of the PknFGST-tagged kinase from *Mtb* were described previously [8].

### Immunoblotting

Bacteria were disrupted by bead beating with 1-mm-diameter glass beads and the total protein concentration in each cell lysate was determined using a bicinchoninic acid (BCA) protein assay reagent kit (Pierce). Equal amounts of proteins (20 µg) were separated on 12% SDS-PAGE gels and were transferred to a nitrocellulose membrane. The membrane was saturated with 1% BSA in PBS/Tween 0.1% and either probed with rat anti-KasA antibodies (dilution1∶500) [54], rabbit anti-AccD6 antibodies (dilution, 1∶1,000) [55] or anti-phosphothreonine antibodies (dilution, 1∶1,000) (Cell Signaling). After washing, the membrane was incubated with either secondary antibodies labeled with IRDye infrared dyes (Odyssey Classic) or alkaline-conjugated anti-rabbit secondary antibodies (dilution, 1∶7,000) and revealed with BCIP/NBT, according to the manufacturer's instructions.

### Mycolic acid extraction and purification

50–100 ml cultures of *Mtb* strains were grown to mid-log phase in Middlebrook 7H9 medium at 37°C. Cells were harvested and FAMEs and MAMEs were extracted as reported [56], subjected to one-dimensional thin layer chromatography (TLC) with silica gel plates (silica gel 60F_254_; Merck, Germany) and developed in hexane/ethyl acetate (19∶1, v/v; 3 runs). Lipids were revealed by spraying the plates with molybdophosphoric acid followed by charring. α-, methoxy- and keto-mycolic acids were then purified using preparative TLC plates and detection by spraying with ethanolic Rhodamine 6G to visualize the lipids under a UV lamp. Areas corresponding to the different mycolic acid subspecies were scrapped off the plates and extracted from the silica gel with diethyl ether. Samples were then resolved again on a second preparative TLC plate and re-extracted. Purity of the mycolates was then assessed on a standard TLC plate in hexane/ethyl acetate (19∶1, v/v; 3 runs) prior to NMR and MALDI-TOF analysis.

### Extraction and analysis of PAT

*Mtb* cultures (10 ml), grown in Middlebrook 7H9-OADC-glycerol-tyloxapol to an OD_600 nm_ of 0.2, were treated with either ^14^C-acetate (10 µCi) or ^14^C-propionate (20 µCi) for 20 h or 2 days, respectively. Apolar lipids were extracted by mixing the cell pellets with methanol (2 ml), 0.3% aqueous NaCl solution (0.2 ml) and petroleum ether (1 ml) for 15 min. The suspensions were centrifuged and the upper petroleum ether phases were removed. The cell pellets were extracted a second time with petroleum ether (1 ml) for 15 min. The petroleum ether phases were combined, dried, and resuspended in dichloromethane (0.2 ml). The same amount of cpm was loaded onto a Silica gel 60 F254 250-µm aluminum plate (10×10 cm) and eluted [1^st^ dimension∶petroleum ether/acetone 23∶2, ×3; 2^nd^ dimension∶toluene/acetone 19∶1]. ^14^C-Radiolabeled species were detected by autoradiography after exposure at −80°C for 2 days on X-ray film.

### NMR analyses

For NMR analyses, MAMEs were dissolved into deuterated chloroform containing 0.01% of TMS and transferred into Shigemi tubes matched for D_2_O. Then 0.1 ml of deuterium oxide was added to avoid solvent evaporation during long acquisition. 1D proton NMR spectra were recorded at 300K on a 400 MHz Avance II Bruker spectrometer equipped with a 5 mm broad-band inverse probe.

### Mass spectrometric analyses

Mass spectrometric analyses of MAMEs were performed on a Voyager Elite reflectron MALDI-TOF mass spectrometer (PerSeptiveBiosystems, Framingham, MA, USA), equipped with a 337 nm UV laser. Samples were solubilized in 1 µl chloroform/methanol (2∶1, v/v) and mixed on target with 1 µl of 2,5-dihydroxybenzoic acid matrix solution (10 mg/ml dissolved in chloroform/methanol 2∶1, v/v).

## Supporting Information

Text S1 Contains supplementary Materials and Methods, supplementary Tables 1–4, supplementary Figures 1–8 and references.
(DOCX)
