# Supplementary material for: Phosphorylation of KasB Regulates Virulence and Acid-Fastness in Mycobacterium tuberculosis
Source: PLoS Pathog. 2014 May 8;10(5):e1004115. doi: 10.1371/journal.ppat.1004115 (PMC4014462; doi:10.1371/journal.ppat.1004115)
Supplement: Text S1 — Contains supplementary Materials and Methods, supplementary Tables 1–4, supplementary Figures 1–8 and references. (DOCX) [file ppat.1004115.s001.docx]

**Phosphorylation of KasB Regulates Virulence and**

**Acid-Fastness in *Mycobacterium tuberculosis***

Catherine Vilchèze^1^, Virginie Molle^2^, Séverine Carrère-Kremer^2^, Jade Leiba^2^, Lionel Mourey^4,5^, Shubhada Shenai^6^, Grégory Baronian^2^, Joann Tufariello^1^, Travis Hartman^1^,

Romain Veyron-Churlet^2, ¶^, Xavier Trivelli^7,8^, Sangeeta Tiwari^1^, Brian Weinrick^1^,

David Alland^6^, Yann Guérardel^7,8^, William R. Jacobs, Jr.^1^ and Laurent Kremer^2,3^

**Supporting information file containing additional materials and methods and references, supporting Tables 1-4 and Supporting Figures 1-8.**

**Materials and Methods S1**

**C-terminus EGFP fusion proteins constructs**

The *egfp* gene was PCR amplified from using the forward primer containing a MscI site 5’-AGCATATGGCCATTAATTCTGCAGTCGACGGTACCG-3’ and the reverse primer containing a HindIII site 5’- GCAAGCTTTTACTTGTACAGCTCGTCCATGCCG-3’ and cloned into the pVV16 vector cut with MscI/HindIII to generate pVV16-EGFP.

The *Rv1818c* gene was amplified from H37Rv genomic DNA using the forward primer containing a NdeI site 5’- AGCATATGTCATTTGTGGTCACGATCC-3’ and the reverse primer containing a BamHI site 5’-ACGGATCCCGGTAACCCGTTCATCCCG-3’, and cloned into the pVV16-EGFP vector cut with the same enzymes to produce pVV16-Rv1818c/EGFP.

The various *kasB* alleles were PCR amplified from pETPhos_*kasB_WT*,pETPhos_*kasB_T334A/T336A* and pETPhos_*kasB_T334D/T336D* using the forward primer containing a NdeI site 5’-TAATAGctCATATGgtgggggtccccccgcttgcg-3’ and the reverse primer containing a BglII site 5’-GAAGATCTGTACCGTCCGAAGGCG -3’, and cloned into the pVV16-EGFP vector cut with NdeI/BamHI.

**Fluorescence microscopy**

*M. smegmatis* mc^2^155 and BCG Pasteur transformants were grown at 37°C in Sauton broth supplemented with 10% OADC until an OD_600_ of 0.9-1.1 was reached. Cells (20µl) were fixed by fire on slides and then mounted using Prolong Gold antifade reagent (Invitrogen). Slides were analyzed on a Zeiss Axioimager, and pictures were captured using Zeiss Axiocam MRm with the Axio Vision Rel. Software (version 4.8).

**Permeability assay**

Permeability was assessed using a previously described method [1]. Briefly, 100 µl of *M. tuberculosis* cultures (~10^7^ cells) were centrifuged for 5 min at 6000 rpm, washed twice in 1 ml PBS, and resuspended in 500 μl PBS containing diethyloxacarbocyanine iodide (DiOC2(3)) (Molecular Probes), and Sytox-Red (Molecular Probes) at final concentrations of 30 μM and 100 nM, respectively. Cells were protected from light and stained at room temperature for 30 min.  Fluorescent signals were acquired on a BD FACSCalibur II in a specialized biosafety cabinet in a BSL-3 containment facility. A normalized permeability value, based on the ratio of [Sytox-Red fluorescence]/[DiOC2(3) green fluorescence], was calculated by adding a constant (96) to the FL-4 value and subtracting the FL-1 value. Analysis was performed in FlowJo (TreeStar Inc.).

**Infection of THP-1 Cells**

THP-1 cells (ATCC#TIB -202), a human acute monocytic leukemia cell line, were grown in suspension using complete RPMI 1640 media supplemented with 10% FBS, 0.5% MEM essential amino acids, 0.5% MEM non essential amino acids, 10 mM HEPES, and 55 µM ß-mercaptoethanol. Cells were maintained and incubated at 37 °C plus 5% CO_2_. For infection experiments with the *M. tuberculosis* strains, THP-1 cells were differentiated to adherent macrophages in complete RPMI containing 50 nM phorbol 12-myristate 13-acetate (PMA, Sigma, MO) for ≥16h prior to infection. 2x10^5^ cells were plated per well in 48 well and infected in complete RPMI containing 10% human serum at Multiplicity of Infection (MOI) of 3:1 (3-bacteria/1 cell) for 4 h. Further, cells were washed 3 times with complete RPMI to remove extracellular bacilli. At various times, the infected cells were lysed in PBS containing 0.05% SDS for 5 min at room temperature to determine the numbers of CFU/ml on Middlebrook 7H10 agar plates.

**References**

1. Snapper SB, Melton RE, Mustafa S, Kieser T, Jacobs WR, Jr. (1990) Isolation and characterization of efficient plasmid transformation mutants of Mycobacterium smegmatis. Mol Microbiol 4: 1911-1919.

2. Canova MJ, Kremer L, Molle V (2008) pETPhos: a customized expression vector designed for further characterization of Ser/Thr/Tyr protein kinases and their substrates. Plasmid 60: 149-153.

3. Molle V, Leiba J, Zanella-Cleon I, Becchi M, Kremer L (2010) An improved method to unravel phosphoacceptors in Ser/Thr protein kinase-phosphorylated substrates. Proteomics 10: 3910-3915.

4. Stover CK, de la Cruz VF, Fuerst TR, Burlein JE, Benson LA, et al. (1991) New use of BCG for recombinant vaccines. Nature 351: 456-460.

5. Jackson M, Crick DC, Brennan PJ (2000) Phosphatidylinositol is an essential phospholipid of mycobacteria. J Biol Chem 275: 30092-30099.

6. Molle V, Brown AK, Besra GS, Cozzone AJ, Kremer L (2006) The condensing activities of the Mycobacterium tuberculosis type II fatty acid synthase are differentially regulated by phosphorylation. J Biol Chem 281: 30094-30103.

7. Kremer L, Douglas JD, Baulard AR, Morehouse C, Guy MR, et al. (2000) Thiolactomycin and related analogues as novel anti-mycobacterial agents targeting KasA and KasB condensing enzymes in Mycobacterium tuberculosis. J Biol Chem 275: 16857-16864.

8. Novo DJ, Perlmutter NG, Hunt RH, Shapiro HM (2000) Multiparameter flow cytometric analysis of antibiotic effects on membrane potential, membrane permeability, and bacterial counts of Staphylococcus aureus and Micrococcus luteus. Antimicrob Agents Chemother 44: 827-834.

9. Shapiro HM (2008) Flow cytometry of bacterial membrane potential and permeability. Methods Mol Med 142: 175-186.

10. Delogu G, Pusceddu C, Bua A, Fadda G, Brennan MJ, et al. (2004) Rv1818c-encoded PE_PGRS protein of Mycobacterium tuberculosis is surface exposed and influences bacterial cell structure. Mol Microbiol 52: 725-733.

**Supporting information legends**

**Table S1.** *M. tuberculosis* strains used in this study.

**Table S2.** Bacterial strains, plasmids and phages used in this study.

**Table S3.** Primers used in this study.

**Table S4.** Sequences of primers and molecular beacons used for quantification of specific RNA products.

**Figure S1. Lack of *in vitro* phosphorylation of KasB phosphoablative (KasB_T334A/T336A) by several kinases.** The soluble domains of seven recombinant *Mtb* STPKs were expressed and purified as GST-tagged fusions and incubated with purified His-tagged KasB_T334A/T336A and [γ-^33^]ATP. Samples were separated by SDS-PAGE, stained with Coomassie Blue (upper panel) and visualized by autoradiography after overnight exposure to a film (lower panel).

**Figure S2. SDS-PAGE analysis of the trypsinolysis kinetics of wild-type and mutated KasB proteins.** The purified KasB His-tagged proteins were obtained by purification by gel filtration (AKTA system, GE Healthcare) in order to eliminate all putative contaminant proteins and diluted to 0.2 mg/mL in buffer (50 mM Tris, pH 7.5, containing 50 mM NaCl and 0.5 mM DTT). After incubation with trypsin (Sigma) to a final concentration of 0.01 mg/mL at 37 °C for 0, 1, 2 , 5, 10 and 30 min, 20 μL aliquots were collected, mixed with SDS-PAGE loading buffer, boiled for 5 min and loaded on a denaturating 15 % acrylamide gel. Molecular weight markers (Invitrogen) are shown (kDa). The rates of trypsinolysis were similar with all three samples.

**Figure S3.** Details of proton NMR spectra in CDCl_3_ recorded at 400 MHz and 300K of purified **(A)** α-MAMEs, **(B)** methoxy-MAMEs and **(C)** keto-MAMEs isolated from the *Mtb* wild-type, Δ*kasB* and KasB_T334D/T336D strains. * TMS and its ^13^C satellites, ** impurity.

**Figure S4. Permeability of *M. tuberculosis kasB* mutants.** *Mtb* strains (~10^7^ cells) were treated with diethyloxacarbocyanine iodide (DiOC2 (3)) and Sytox-Red. Fluorescent signals were acquired on a BD FACSCaliburII. Permeability was assessed as described in [1,2], based on the ratio of [Sytox-Red fluorescence]/[DiOC2 (3) green fluorescence]. The Sytox-Red fluorescence signal increases when a membrane is more permeable.

**Figure S5. Intracellular survival and uptake of the various *M. tuberculosis* strains by human THP-1 macrophages. (A) Growth of *M. tuberculosis kasB* mutant strains in THP-1 macrophages.** An MOI of 3 was used to infect THP-1 cells which were lysed after 4 h, 1, 2, 3, and 6 days post-infection. CFU/ml were determined by plating dilutions onto supplemented Middlebrook 7H10 plates. **(B) Uptake of KasB strains in THP-1 cells.** Uptake was determined as the number of bacteria in the macrophages after 4 hours of infection divided by the initial inoculum. The experiment was done in duplicate. Blue, *M. tuberculosis* CDC1551; green, *M. tuberculosis* KasB_T334A/T336A; black, *M. tuberculosis* Δ*kasB*; black with white stripes, *M. tuberculosis* Δ*kasB* pMV261-*kasB*; red, *M. tuberculosis* KasB_T334D/T336D; red with white stripes, *M. tuberculosis* KasB_T334D/T336D pMV261-*kasB*.

**Figure S6. Transcriptional profile of the *M. tuberculosis* KasB mutants.** Annotated genes up-regulated **(left panel)** or down-regulated **(right panel)** 2-fold or more are shown. Classification (Cl), based on the Tuberculist website (<http://genolist.pasteur.fr/TubercuList/>), stands for: 0, virulence, detoxification, adaptation; 1, lipid metabolism; 2, information pathways; 3, cell wall and cell processes; 5, insertion sequences and phages; 6, PE/PPE; 7, intermediary metabolism and respiration; 9, regulatory proteins; 10, conserved hypotheticals. Highlighted in grey are genes that are specifically up- or down-regulated in the KasB phosphomimetic mutant. NaN: not detected. *: not significant by Significance Analysis of Microarrays (SAM) with a False Discovery Rate (FDR) < 5%. All of the genes involved in PAT biosynthesis meet this significance threshold.

**Figure S7. Fluorescence analysis of mycobacterial strains growing in liquid medium and analyzed by fluorescence microscopy.** *M. smegmatis* mc^2^155 and BCG Pasteur were transformed with the various KasB mutant proteins fused to EGFP. Strains transformed with pVV16-EGFP alone were used as a control where GFP is uniformly expressed in the cytoplasm without any sign of compartmentalization. *M. smegmatis* expressing a Rv1818c/PE_PGRS33 GFP-tagged protein indicated that this protein was localized in the mycobacterial cell wall, mostly at the bacterial cell poles, as previously described [3]. Similar results were obtained with *M. smegmatis* or *M. bovis* BCG expressing the different KasB/EGFP fusion proteins. Overall, these data suggest that phosphorylation of KasB does not affect the polar localization of the protein.

**Figure S8. PDIM profile in the various *M. tuberculosis* KasB mutant strains.** Autoradiographs of thin layer chromatograms of apolar lipids derived from [1-^14^C]-propionate labeling in the various *Mtb* KasB strains. Total lipids (1000 counts) were loaded on TLC plates and developed thrice in petroleum ether/ethyl acetate (98:2, v/v) in the first direction and once in petroleum ether/acetone (98:2, v/v) in the second direction.

**Table S1.** *M. tuberculosis* strains used in this study.

**Strain Allele *kasB* allele How constructed Source**

**number**

CDC1551 *kasB* Wild-Type CSU^a^

mc^2^5860 *kasB5* Δ*kasB* ST^b^ of CDC1551 with phAE801 This work

mc^2^5862 *kasB6* T334A/T336A ST of CDC1551 with phAE802 This work

mc^2^5864 *kasB7* T334D T336D ST of CDC1551 with phAE803 This work

a: Colorado State University

b: specialized transduction

**Table S2.** Bacterial strains, plasmids and phages used in this study.

| **Strains/Plasmids/Phages** | **Genotype or Description** | **Source or Reference** |
| --- | --- | --- |
| *E. coli* TOP10 | F^-^ *mcr*A Δ(*mrr*-*hsd*RMS-*mcr*BC) φ80*lacZΔM15* Δ*lacX74 deoR recA1 araD139* Δ(*ara*-*leu*)7697 *galU galK rpsL endA1 nupG*; used for general cloning | Invitrogen |
| *E. coli* BL21(DE3)Star | F2 *ompT hsdSB*(rB2 mB2) *gal dcm* (DE3); used to express recombinant proteins in *E. coli* | Stratagene |
| *M. smegmatis* mc^2^155 | *ept-1* | [4] |
| *M. bovis* BCG 1173P2 | Vaccine strain | WHO, Stockholm |
| *M. bovis* BCG Δ*kasB* | *M. bovis* BCG 1173P2 with deleted *kasB* gene | This work |
| pETPhos | pET15b (Novagen) derivative including the replacement of the thrombin site coding sequence with a tobacco etch virus (TEV) protease site and Ser to Gly mutagenesis in the Nterm His-tag | [5] |
| pETPhos_*kasB_WT* | pETPhos derivative used to express His-tagged fusion of WT KasB in *E.coli* | This work |
| pETPhos_*kasB_T334A/T336A* | pETPhos derivative used to express His-tagged fusion of KasB_T334A/T336A in *E.coli* | This work |
| pETPhos_*kasB_T334D/T336D* | pETPhos derivative used to express His-tagged fusion of KasB_T334D/T336D in *E.coli* | This work |
| pETDuet | *E. coli* expression vector allowing to co-express a kinase (PknF) with its substrate | [6] |
| pETDuet_*kasB* | pETDuet derivative, allows to co-express PknF with KasB | This work |
| pVV16 | *E. coli*/mycobacterial shuttle vector, allows expression of C-term His-tagged proteins, derived from pMV261 [7] | [8] |
| pVV16_*kasB_WT* | pVV16 derivative used to express His-tagged fusion of WT KasB in mycobacteria | [9] |
| pVV16_*kasB_T334A/T336A* | pVV16 derivative used to express His-tagged fusion of KasB_T334A/T336A in mycobacteria | This work |
| pVV16_*kasB_T334D/T336D* | pVV16 derivative used to express His-tagged fusion of KasB_T334D/T336D in mycobacteria | This work |
| pMV261::*kasB* | *M.tuberculosis kasB* cloned downstream of  the *hsp60* promoter of the *E. coli*-mycobacteria shuttle plasmid vector pMV261 | [10] |
| pYUB1471 | Allelic Exchange Substrate (AES) cloning vector for specialized transduction | Paras and Jacobs, manuscript in preparation |
| pYUB1471_*kasB_ T334A/T336A* | AES vector for specialized transduction of *kasB_ T334A/T336A* | This work |
| pYUB1471_*kasB_ T334D/T336D* | AES vector for specialized transduction of *kasB_ T334D/T336D*  p004S p004S_*kasB* _*kasB* | This work |
| pYUB1471_Δ*kasB* | AES vector for specialized transduction for deletion of *kasB* | This work |
| phAE801 | phAE159 containing pYUB1471_Δ*kasB* | This work |
| phAE802 | phAE159 containing pYUB1471_*kasB_ T334A/T336A* | This work |
| phAE803 | phAE159 containing pYUB1471_*kasB_ T334D/T336D* | This work |

**Table S3.** Primers used in this study.

| **Primers** | **5' to 3' Sequence^ab^** |
| --- | --- |
| Nterm KasB | taa tag ctc ata tgg tgg ggg tcc ccc cgc ttg cg (NdeI) |
| Cterm KasB | taa tag ctg cta gct tag tac cgt ccg aag gcg att gc (NheI) |
| KasB T334A dir | cca cgt caa tgc gca cgc c**g**c cgg cac cca ggt cgg cga c |
| KasB T334A rev | gtc gcc gac ctg ggt gcc gg**c** ggc gtg cgc att gac gtg g |
| KasB T336A dir | caa tgc gca cgc cac cgg c**g**c cca ggt cgg cga cct ggc c |
| KasB T336A rev | ggc cag gtc gcc gac ctg gg**c** gcc ggt ggc gtg cgc att g |
| KasB T334A/6A dir | caa tgc gca cgc c**g**c cgg c**g**c cca ggt cgg cga cct ggc c |
| KasB T334A/6A rev | ggc cag gtc gcc gac ctg gg**c** gcc gg**c** ggc gtg cgc att g |
| KasB T334D/6D dir | caa tgc gca cgc c**ga** cgg c**ga** cca ggt cgg cga cct ggc c |
| KasB T334/6D rev | ggc cag gtc gcc gac ctg g**tc** gcc g**tc** ggc gtg cgc att g |
| LL2 | TTTTTTTTCAGAAACTGAGG AACTGGTCTTCAGTTAC (AlwNI) |
| LR1 | TTTTTTTTCAGTTCCTGTTAG TACCGTCCGAAGGCGA (AlwNI) |
| RL | TTTTTTTTCCATAGATTGGCCAGCGTTA CGCGACAGGAG (Van91I) |
| RR | TTTTTTTTCCATCTTTTGGACCACCA CCAGCGGAATCCC (Van91I) |
| kasB_F | CGTTACTGAAGCACGACATC |
| kasB_R | GTACCGTCCGAAGGCGATTG |

^a^ Restriction sites are underlined and specified into brackets.

^b^ Mutagenized bases are shown in bold.

**Table S4.** Sequences of primers and molecular beacons used for quantification of specific RNA products.

| **Target** | **RT and real time PCR primer sequences**  **(5’ to 3’)^a^** | **Molecular beacon sequence**  **(5’ to 3’)^b,c^** |
| --- | --- | --- |
| ***kasB*** | RT primer: GAA CAC AAA GCC GTC GCG GTC CC  F: CCA TCG CCG GGT TCG CTC AGA  R: CGC GGT CCC TGT CGA ATG GGC | **CCACG**CACCAACAACGACGACCCCGCCG **CGTGG**^b^ |
| ***cmaA2*** | RT primer: GAA CTT GAT GAA GCG CAG CAG GC  F: CTG CAC ACC ATC ACT ATC CCG GA  R: GCA GGC TCA TCG GAG ACG TCA AG | **ACGC**CAAAGAGGAAGCCCAGGAGCTG**GCGT**^b^ |
| ***sigA*** | RT primer: ATC TGG CGG ATG CGT TCC CGG  F: GGC CAG CCG CGC ACC CTT GAC  R: CGG ATG CGT TCC CGG GTC ACG | **CGC**ACGAGATCGGCCAGGTCTACGGCGT**GCG^c^** |
| ***16S rRNA*** | RT primer: GCC GGA CAC CCT CTC AGG CC  F: CGC TTT AGC GGT GTG GGA TGA  R: GCC GGC TAC CCG TCG TCG CC | **ACG**CCCGCGGCCTATCAGCTTGTTGG**GCGT^c^** |
| ***rrnAP1*** | RT primer: TTC TCA AAC AAC ACG CTT GCT TG  F: CCT ATG GAT ATC TAT GGA TGA C  R: GCA ACC CTG CCA GTC TAA TAC AA | **ACGC**CCTGTTCTTGACTCCATTGCCGG**G CGT** |

^a^RT, Reverse Transcription; F, Forward; R, Reverse. ^b^Molecular beacons labelled on the 5’ end with 5-carboxyfluoroscein (FAM) and on the 3’ end with DABCYL. ^c^Molecular beacons labelled on the 5’ end with tetrachloro-6-carboxyfluorescein (TET) and on the 3’ end with DABCYL.

**Figure S1**

**PknL**

**M (kDa)**

**PknF**

**PknA**

**PknD**

-

**PknH**

**PknE**

**PknB**


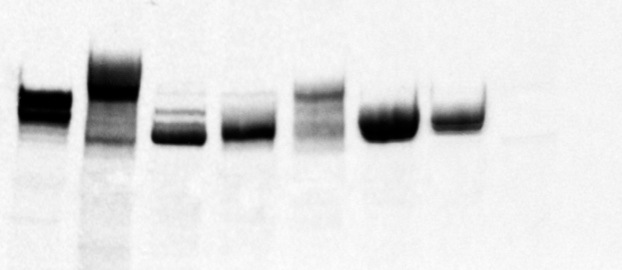

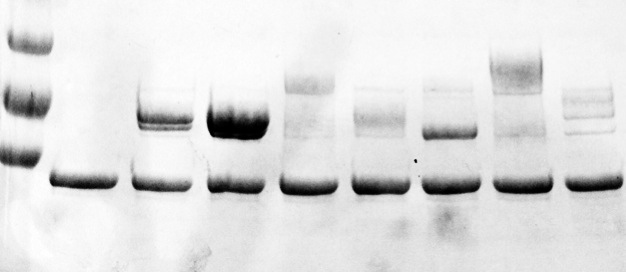


**Autoradiogram**

**Coomassie**

95 —

72 —

55 —

kinases

KasB_T334A/T336A

kinases

KasB_T334A/T336A

95 —

72 —

55 —

**Figure S2**

55 —

36 —

72 —

**M (kDa)**

**KasB_WT**

**KasB_T334A/T336A**

**Incubation time with trypsin (min)**

**0 1 2 5 10 30 0 1 2 5 10 30 0 1 2 5 10 30**

28 —

17 —

**KasB_T334D/T336D**


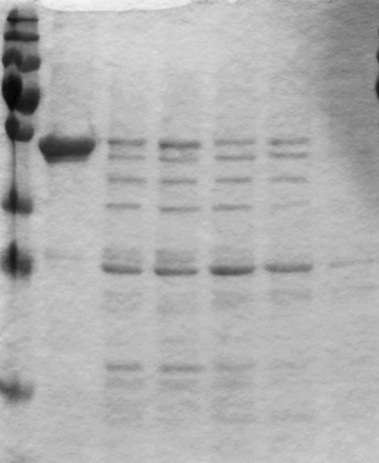

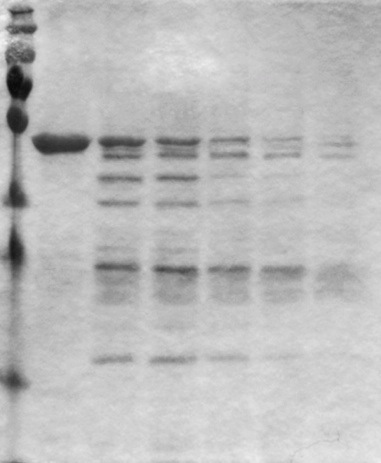

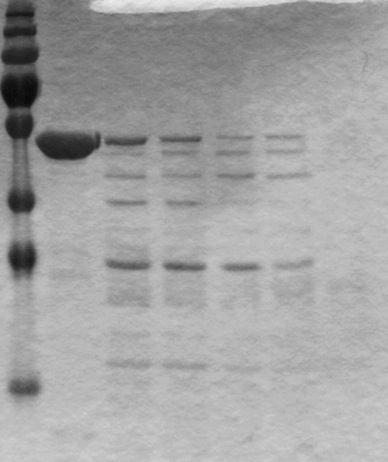


**Figure S3**

**
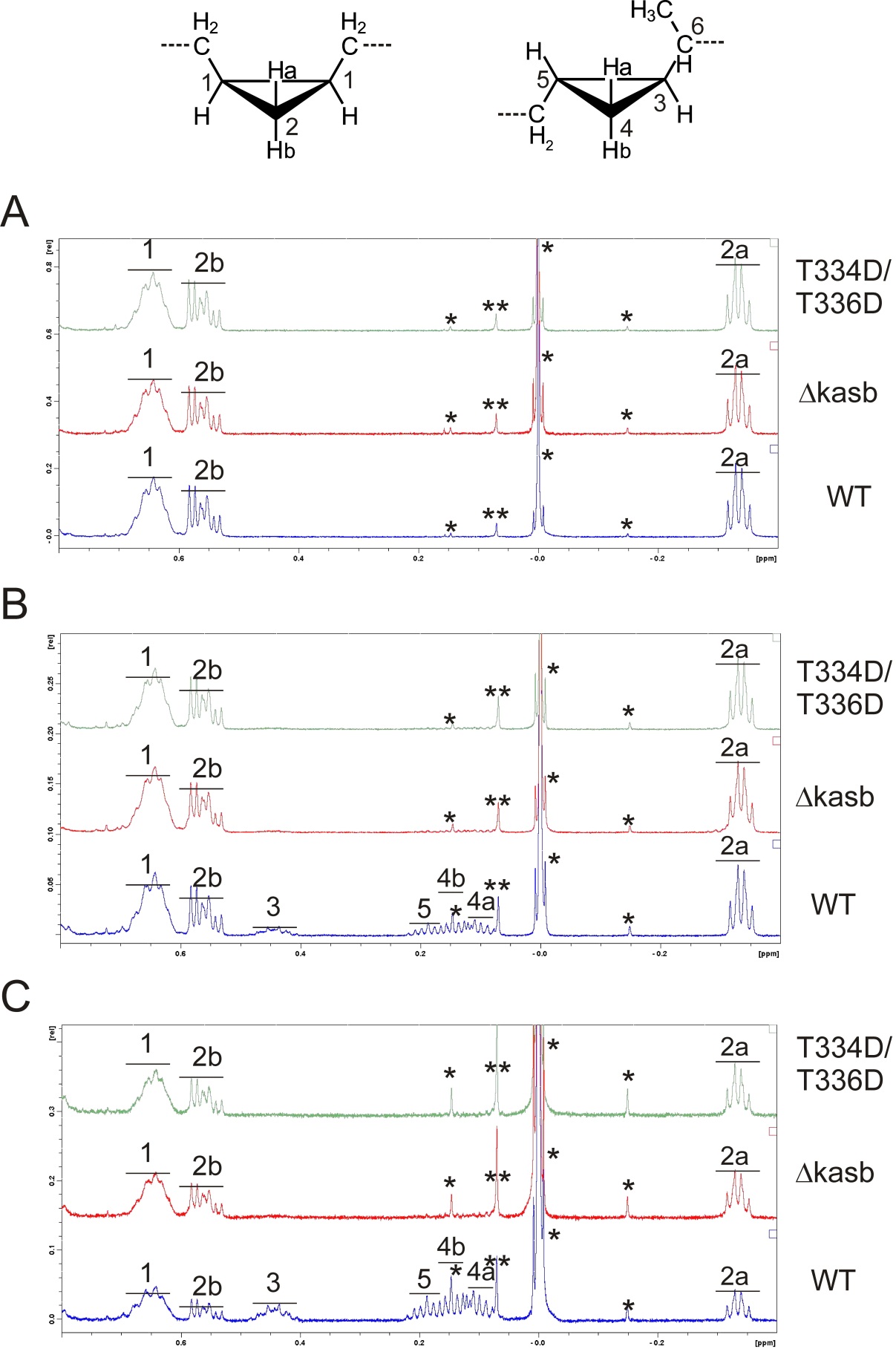
**

**Figure S4**


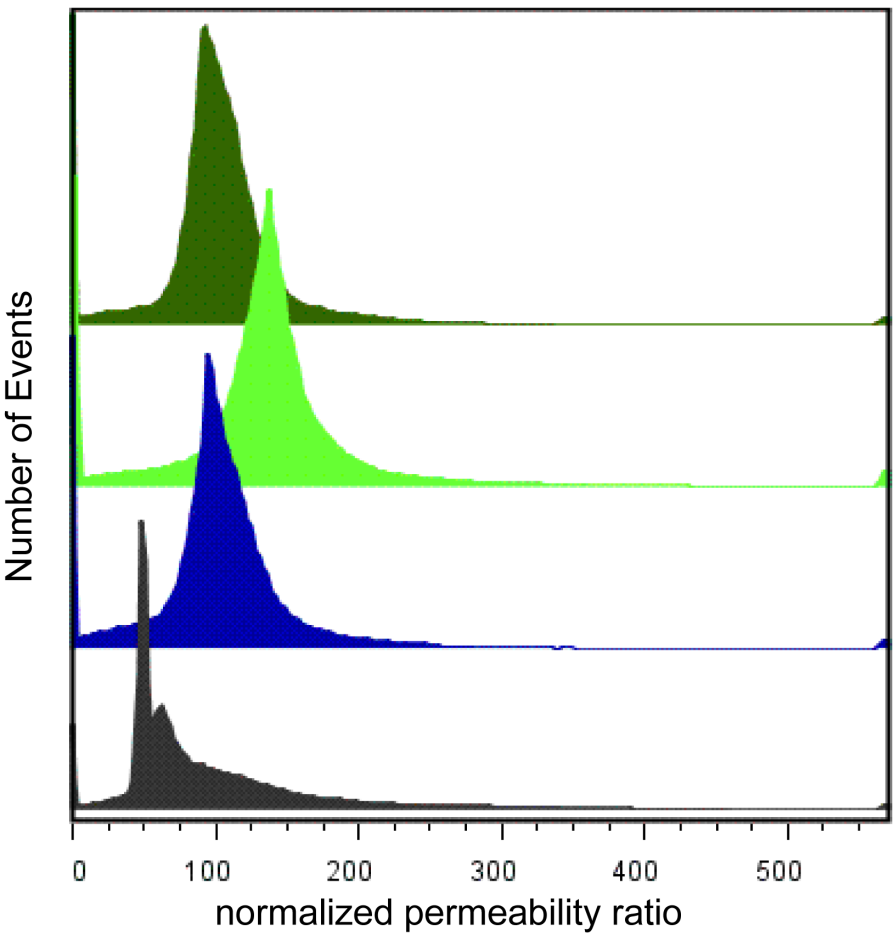


parental

KasB_334A/336A

KasB_334D/336D

Δ*kasB*

**Figure S5**

**
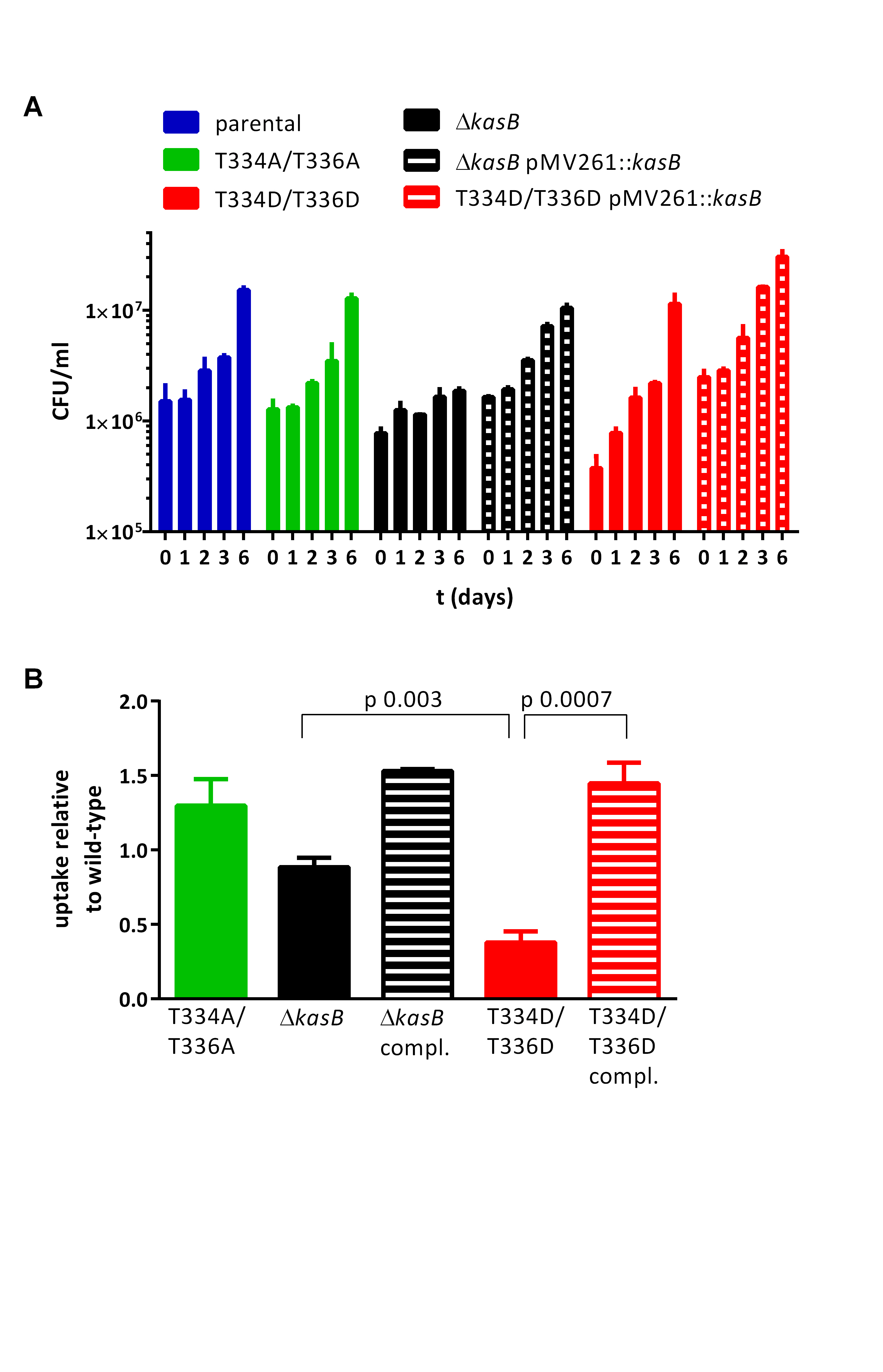
**


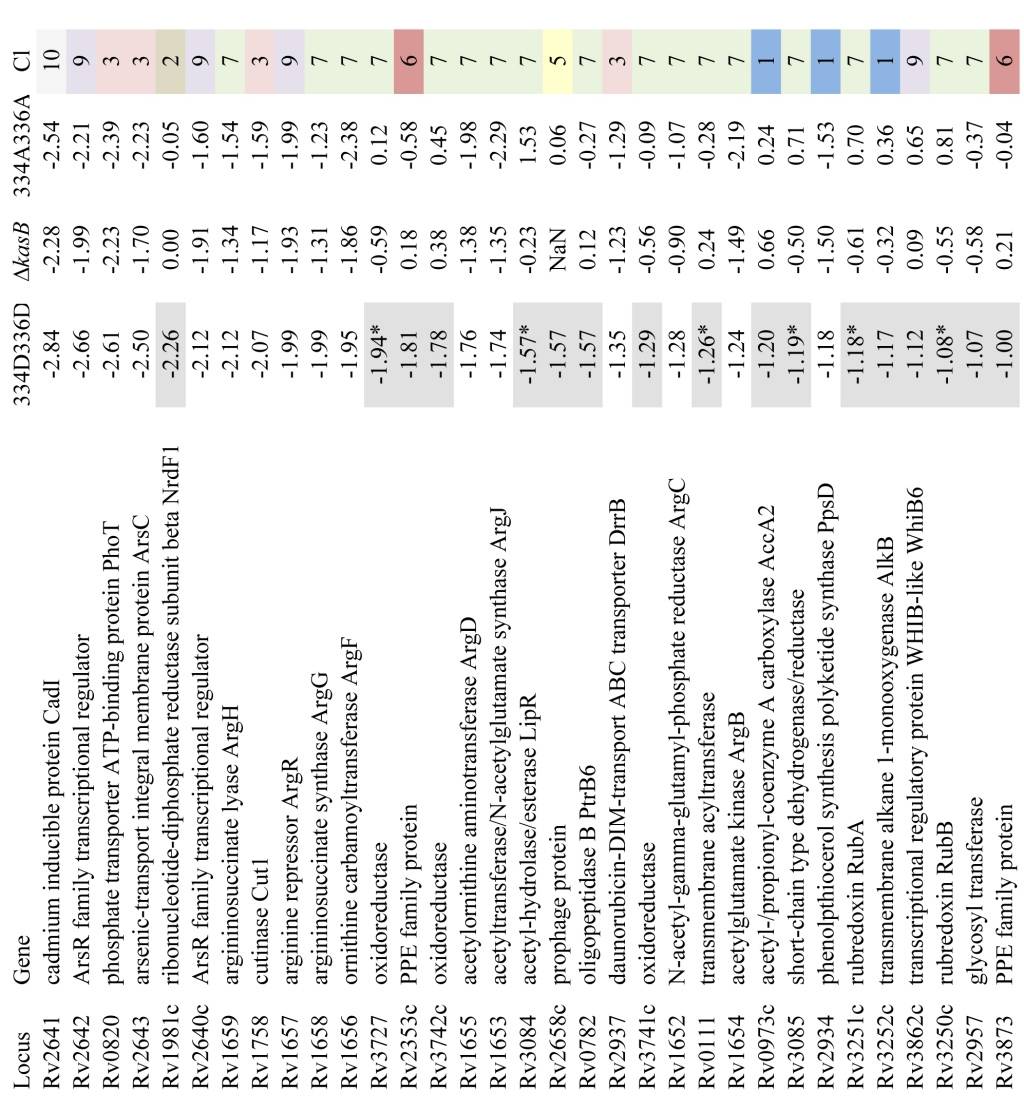


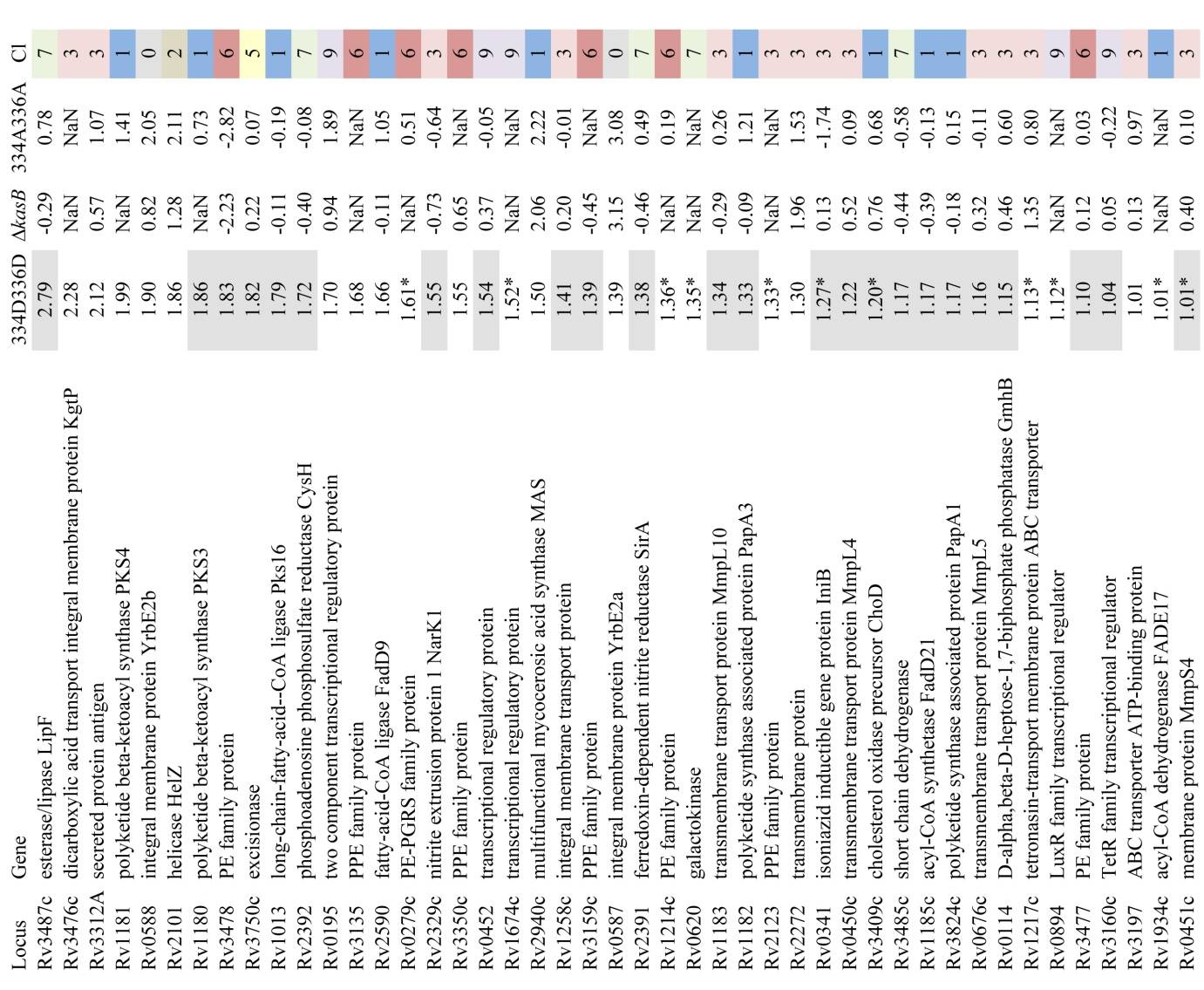


**Figure S6**

**Figure S7**

**Figure S8**

**References**

1. Shapiro HM (2008) Flow cytometry of bacterial membrane potential and permeability. Methods Mol Med 142: 175-186.

2. Novo DJ, Perlmutter NG, Hunt RH, Shapiro HM (2000) Multiparameter flow cytometric analysis of antibiotic effects on membrane potential, membrane permeability, and bacterial counts of Staphylococcus aureus and Micrococcus luteus. Antimicrob Agents Chemother 44: 827-834.

3. Delogu G, Pusceddu C, Bua A, Fadda G, Brennan MJ, et al. (2004) Rv1818c-encoded PE_PGRS protein of Mycobacterium tuberculosis is surface exposed and influences bacterial cell structure. Mol Microbiol 52: 725-733.

4. Snapper SB, Melton RE, Mustafa S, Kieser T, Jacobs WR, Jr. (1990) Isolation and characterization of efficient plasmid transformation mutants of Mycobacterium smegmatis. Mol Microbiol 4: 1911-1919.

5. Canova MJ, Kremer L, Molle V (2008) pETPhos: a customized expression vector designed for further characterization of Ser/Thr/Tyr protein kinases and their substrates. Plasmid 60: 149-153.

6. Molle V, Leiba J, Zanella-Cleon I, Becchi M, Kremer L (2010) An improved method to unravel phosphoacceptors in Ser/Thr protein kinase-phosphorylated substrates. Proteomics 10: 3910-3915.

7. Stover CK, de la Cruz VF, Fuerst TR, Burlein JE, Benson LA, et al. (1991) New use of BCG for recombinant vaccines. Nature 351: 456-460.

8. Jackson M, Crick DC, Brennan PJ (2000) Phosphatidylinositol is an essential phospholipid of mycobacteria. J Biol Chem 275: 30092-30099.

9. Molle V, Brown AK, Besra GS, Cozzone AJ, Kremer L (2006) The condensing activities of the Mycobacterium tuberculosis type II fatty acid synthase are differentially regulated by phosphorylation. J Biol Chem 281: 30094-30103.

10. Kremer L, Douglas JD, Baulard AR, Morehouse C, Guy MR, et al. (2000) Thiolactomycin and related analogues as novel anti-mycobacterial agents targeting KasA and KasB condensing enzymes in Mycobacterium tuberculosis. J Biol Chem 275: 16857-16864.
